# Four- and five-component molecular solids: crystal engineering strategies based on structural inequivalence

**DOI:** 10.1107/S2052252515023945

**Published:** 2016-01-05

**Authors:** Niyaz A. Mir, Ritesh Dubey, Gautam R. Desiraju

**Affiliations:** a Solid State and Structural Chemistry Unit, Indian Institute of Science, Bangalore 560 012, India

**Keywords:** crystal engineering, intermolecular interactions, polymorphism, cocrystals, crystal design

## Abstract

A synthetic strategy is described for the co-crystallization of four- and five-component molecular crystals, based on the fact that if any particular chemical constituent of a lower cocrystal is found in two different structural environments, these differences may be exploited to increase the number of components in the solid. 2-Methylresorcinol and tetramethylpyrazine are basic template molecules that allow for further supramolecular homologation. Ten stoichiometric quaternary cocrystals and one quintinary cocrystal with some solid solution character are reported. Cocrystals that do not lend themselves to such homologation are termed synthetic dead ends.

## Abstract 2

A logic driven synthetic approach is used in this first report of the isolation of stoichiometric four-component molecular solids. A possible extension to a five-component solid is also described.

## 1. Introduction   

Retrosynthetic analysis with supramolecular synthons remains at the cornerstone of logic driven crystal engineering (Desiraju, 1995 ▸). Supramolecular synthons encapsulate critical shape and chemical recognition information and are the structural units that persist through all stages of crystallization (Desiraju, 1997*a* ▸; Desiraju, 2007 ▸). Crystal engineering is a form of solid state supramolecular synthesis (Desiraju, 1997*b* ▸) and the complexity of a synthetic target is assessed in terms of precise orientations and topologies of specified chemical constituents, and the eventual aim is a crystal architecture with pre-desired properties (Desiraju *et al.*, 2011 ▸). Crystallization, since antiquity, has been a method of purification and it is an excluding rather than an including phenomenon. Generally speaking, when a mixture of compounds is taken for crystallization, the outcome is a single component crystal. Multi-component crystals are more difficult to obtain, and their isolation is often unpredictable. The formation of a two-component crystal **AB** from **A** and **B** implies that interactions of the type **A**⋯**B** are better in some respect than interactions of the type **A**⋯**A** or **B**⋯**B**, with the caveat that these interactions involve either or both shape and chemical recognition. Therefore, obtaining a multi-component crystal means that one is able to calibrate and assess intermolecular interactions rather carefully. These multi-component crystals (Bond, 2007 ▸), also called cocrystals, have been known since the isolation of quinhydrone (Wöhler, 1844 ▸). Binary cocrystals are of importance in the pharmaceutical industry (Almarsson & Zaworotko, 2004 ▸; Wouters & Quéré, 2012 ▸; Stahly, 2009 ▸). Making a binary cocrystal is now well within the scope of crystal engineering (Desiraju *et al.*, 2011 ▸).

Increasing the number of components in a cocrystal is a crystal engineering equivalent of synthetic complexity (Aakeröy *et al.*, 2001 ▸, 2005 ▸). This is because when one cocrystallizes compounds such as **A**, **B** and **C**, one may well obtain binary cocrystals **AB**, **BC**, **AC** rather than the desired ternary **ABC**. Design strategies for ternary cocrystals are based on exploiting chemical differences in the selected molecules (Bučar *et al.*, 2012 ▸; Seaton *et al.*, 2013 ▸; Chakraborty *et al.*, 2014 ▸; Dobrowolski *et al.*, 2014 ▸; Bolla & Nangia, 2015 ▸). Ditopic hydrogen bond donors and acceptors may be manipulated so that a hierarchy of hydrogen bonds emerge, facilitating the formation of ternary cocrystals (Dubey & Desiraju, 2014 ▸, 2015 ▸; Mir *et al.*, 2015 ▸). Alternatively, interactions of different strengths or types may be employed so that a hierarchy is established (Chakraborty *et al.*, 2014 ▸; Tothadi & Desiraju, 2013 ▸). Strong hydrogen bonds, weak hydrogen bonds, halogen bonds, π⋯π interactions and van der Waals interactions may be used in a modular and pre-determined manner to give an interaction orthogonality that is sufficient to form ternary cocrystals. Typically, in an assembly of the type **A**⋯**B**⋯**C**, the interactions **B**⋯**C** could be weaker than the interaction **A**⋯**B** while an interaction of the type **A**⋯**C** may be so feeble that it need not be considered (Bolla & Nangia, 2015 ▸). If **B**⋯**C** is sufficiently close in effectiveness to **A**⋯**B** then ternary **A**⋯**B**⋯**C** would be preferred to binaries **A**⋯**B** and **B**⋯**C**. This strategy is surprisingly effective and we (Chakraborty *et al.*, 2014 ▸; Dubey & Desiraju, 2014 ▸, 2015 ▸; Mir *et al.*, 2015 ▸; Tothadi & Desiraju, 2013 ▸) and others (Aakeröy *et al.*, 2001 ▸, 2005 ▸; Seaton *et al.*, 2013 ▸) have reported cases where ternaries are obtained exclusively in the absence of binaries. Certain techniques of ‘supramolecular homologation’ may also be used, for example, a diacid may be inserted into an amide⋯amide homosynthon without interfering with other interactions in the system (Tothadi & Desiraju, 2013 ▸). This would reliably increase the number of components in a system by one. Shape and chemical arguments may be used together and provide greater control (Bhogala *et al.*, 2005 ▸; Moorthy *et al.*, 2010 ▸; Tothadi *et al.*, 2011 ▸). Host–guest compounds with cavities of different sizes could accommodate guests of specific sizes.

One may ask whether stoichiometric four- and five-component cocrystals can even be prepared given the large number of crystallization possibilities that would seem to be available. In this study, a four-component crystal is taken as one in which four solid components are crystallized together to obtain a single crystalline product that contains all four compounds in a stoichiometric ratio. Such a definition excludes, for example, solvates/hydrates in which the solvent is the third and/or the fourth component and in which introduction of the solvent was not deliberately engineered or even anticipated (this is mostly true of water which need not even be taken as a crystallizing solvent but can still appear in the final crystal) (Clarke *et al.*, 2012 ▸), and also solid solution type entities which are obtained by statistical crystallization techniques (Bhogala & Nangia, 2008 ▸). With such a definition, cocrystallization of a mixture of four compounds could, in principle, give not only binaries but also a number of ternaries. In the end, it would appear that the level and degree of control to make a quaternary cocrystal is formidable.

## 2. Experimental   

Single-crystal X-ray data for all the crystals were collected on a Rigaku Mercury 375/M CCD (XtaLAB mini) diffractometer using graphite monochromator Mo *K*α radiation at 150 K and were processed with *CrystalClear* software (Rigaku, 2009 ▸). Some datasets were collected on a Bruker D8 Quest diffractometer equipped with an Oxford cryosystems N_2_ open-flow cryostat using Mo *K*α radiation. Data integration and data reduction were carried out with the *SAINT-Plus* program (Bruker, 2006 ▸). Structure solution and refinement were performed using *SHELX*2013 (Sheldrick, 2015 ▸) embedded in the *WinGX* suite (Farrugia, 1999 ▸). All non-H atoms were refined anisotropically by the full-matrix least-squares method. H atoms were fixed on the riding model and some of the acidic H atoms were located *via* Fourier maps. *Mercury* Version 3.5 (Macrae *et al.*, 2008 ▸) was used for molecular representations and packing diagrams.

For crystallization, liquid assisted or solvent assisted grinding procedures were employed. In this method the solid components to be crystallized are taken together in definite stoichiometric ratios in a mortar along with few drops of a solvent. The mixture is then ground with a pestle and the process is repeated 2–3 times to get a homogenous mixture. The solid mixture is then taken for crystallizations in different solvents. Detailed crystallization descriptions have been provided in the supporting information.

## 3. Results and discussion   

**Figure 1:**
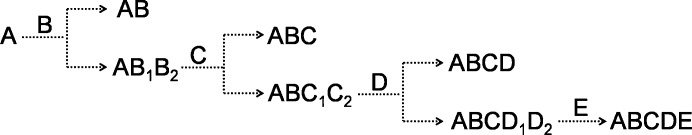
Schematic representation for the synthesis of multi-component crystals. Here, **A**, **B**, **C**, **D** and **E** refer to the molecule in distinct crystal environments. **B_1_, B_2_** refer to the breakdown of structural equivalence at site **B**, and similarly for sites **C** and **D**.

**Figure 2:**
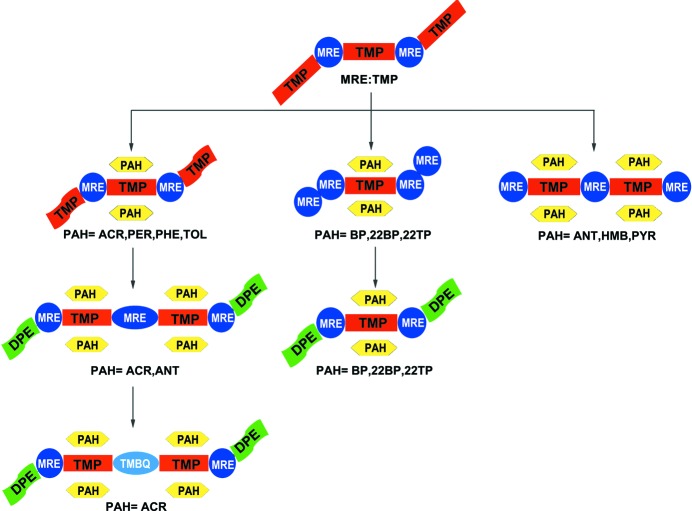
Cartoon representation of crystal synthesis for multi-component crystals. Here, color coding and shapes represent distinct chemical and geometrical features of the molecules.

This letter describes a concise synthetic strategy to increase the number of components in a crystal from one to two (Mir *et al.*, 2015 ▸), to three, to four and eventually to five. The strategy is outlined schematically in Fig. 1 ▸ and in cartoon fashion in Fig. 2 ▸. In Fig. 1 ▸ and henceforth in this paper the letters **A**, **B**, **C**, **D** and **E** refer to molecules in certain crystal environments rather than to the compounds themselves. The strategy is based on the fact that if any particular component in a cocrystal is found in two different environments, these differences may be exploited to increase the number of components. Molecules **A** and **B** are chosen so that two types of binaries are obtained. In the first, the same chemical functionalities in **A** or **B** are located in the same crystal environment and are not susceptible to any further supramolecular differentiation. In the second type, however, the same functional groups in say **B** are found in two types of crystal environments which we shall refer to as **B_1_** and **B_2_**, or they are potentially capable of such differentiation (Smolka *et al.*, 1999 ▸). These crystal environments (or potential environments) may be sufficiently distinct so that a new entity **C** that is introduced will be able to discriminate between the sites and replace just one of **B_1_** or **B_2_**, to give a ternary. These ternaries may be of two types: in the first, the three components **A**, **B** and **C** are found in a single crystallographic environment each and in the second, one of the components, say **C** is found in two slightly different crystallographic environments. We shall refer to these as **ABC** and **ABC_1_C_2_**, respectively. So, in the next step, if a fourth component **D** is taken for the crystallization experiment there is a possibility of obtaining a quaternary **ABCD**. Finally, the same strategy may be employed in the favorable case where **ABCD_1_D_2_** is obtained, to arrive at a quintinary cocrystal **ABCDE**. This design strategy is illustrated in this research letter with the prototype 2-methylresorcinol (MRE).

**Figure 3:**
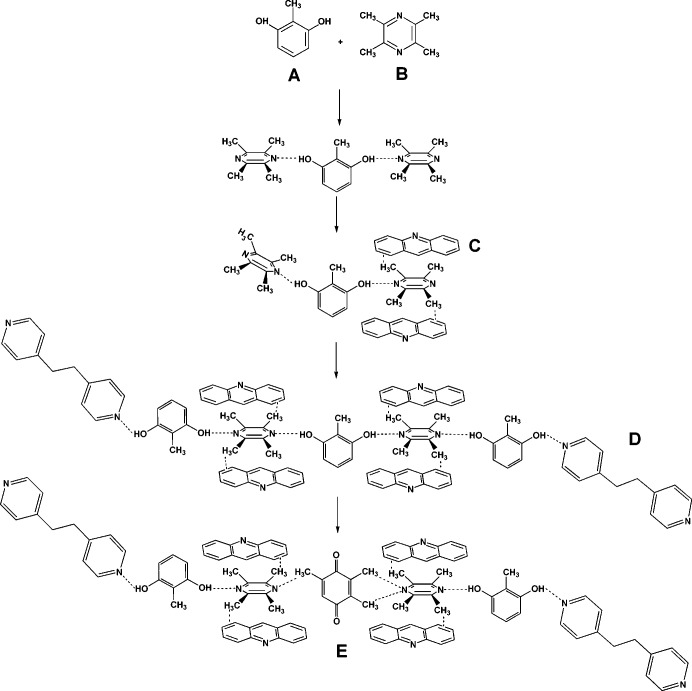
Synthetic scheme for multicomponent crystals. **A**, **B**, **C**, **D** and **E** are representative molecules. Note the systematic insertion of components in each step using structural differentiations available in the crystal.

**Figure 4:**
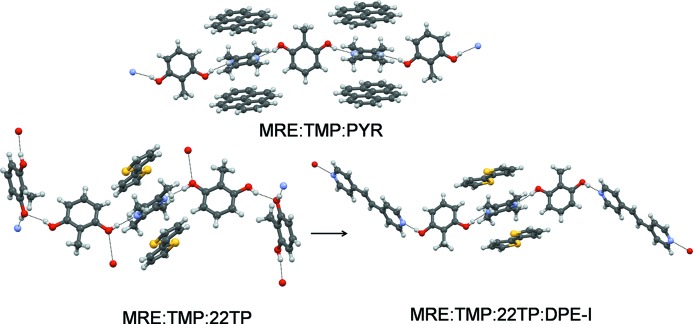
Quaternary cocrystal. The MRE:TMP:PYR ternary solid (top) is a synthetic dead end. The MRE:TMP:22TP:DPE-I quaternary solid may be considered as a development of the MRE:TMP:22TP ternary (bottom) in which the one of the MREs is replaced by the fourth component DPE-I using O—H⋯N hydrogen bonding.

We have reported binary and ternary cocrystals of MRE (Mir *et al.*, 2015 ▸). Here (Fig. 3 ▸) we have selected tetramethylpyrazine (TMP) as a coformer that provides an O—H⋯N mediated 1:1 MRE:TMP cocrystal of the type **AB_1_B_2_** (the two O—H⋯N metrics are different, for example). This lack of structural equivalence has been previously utilized in the synthesis of hydrogen-bonded ternary solids **ABC**, for instance in the 2:1:2 solid MRE·TMP·4DMAP (4DMAP is 4-dimethylaminopyridine). Here, we preferred to modulate interaction strength and selected flat aromatic molecules (PAH) that can form weak C—H⋯π interactions with the methyl groups of TMP. For example, an equimolar ratio of pyrene (PYR) as a template with MRE and TMP provides a stoichiometric 1:1:1 MRE:TMP:PYR ternary solid in which each molecule occupies its own distinct crystal environment. We extended the generality of this strategy with anthracene (ANT) and hexamethylbenzene (HMB) and isolated 1:1:1 MRE:TMP:ANT and 1:1:1 MRE:TMP:HMB ternary solids, respectively (Fig. 4 ▸). These crystal structures follow from a situation wherein O—H⋯N hydrogen-bond inequivalences in the binary MRE:TMP cocrystal are exploited to achieve incorporation of the third component. As mentioned by us previously, the ternaries thus obtained were largely uncontaminated by binaries (as monitored with powder X-ray diffraction).

However, this is not the only type of ternary cocrystal that is obtained. Figs. 2 ▸ and 4 ▸ show two other types: biphenyl (BP), 2,2′-bipyridine (22BP) and 2,2′-bisthiophene (22TP) yield 1:1:1 solids with MRE and TMP with O—H⋯O hydrogen bonds between MRE molecules; acridine (ACR), perylene (PER), phenazine (PHE) and tolan (TOL) give ternaries wherein the ditopic MRE and TMP form an infinite O—H⋯N pattern. However, only every alternate molecule of TMP is involved in C—H⋯π stacking with the third component. An important difference between these two types of ternary and the PYR type is that the PYR type does not lend itself to upgradation into a quaternary (Fig. 4 ▸). There is no inequivalence at any of the three sites **A**, **B** or **C**. It is a synthetic dead end. In the ACR and 22TP types, however, there is a differentiation of structural sites: in the ACR group only one of the TMP molecules is stacked with the PAH; in the 22TP type, MRE forms O—H⋯N and O—H⋯O hydrogen bonds at different sites. Accordingly, these inequivalences may be likened to **C_1_** and **C_2_** in the scheme shown in Fig. 1 ▸. So, we could replace the (unstacked) TMP in the ACR group with another ditopic acceptor such as 1,2-bis(4-pyridyl)ethylene (DPE-I). Using the 22TP ternary as a conceptual starting point for a quaternary, we are effectively replacing an O—H⋯O hydrogen bond with an O—H⋯N by using DPE-I or DPE-II. We obtained six four-component cocrystals **ABCD** with DPE-I or the nearly similar 1,2-bis(4-pyridyl)ethane (DPE-II) as the fourth component and each of BP, 22BP and 22TP as the third component. Typical examples are the 2:1:1:1 solids MRE:TMP:22TP:DPE-I and 2:1:1:1 MRE:TMP:BP:DPE-II, which is **ABCD** in Fig. 1 ▸. *This is the first report in which four solids are taken together for crystallization and the product is a single phase that contains all four chemical species in a fixed stoichiometry.*

Four more quaternaries were obtained in which the crystal structures arise from the ACR group of ternaries. These solids are derived from the 1:2:1 MRE:TMP:ACR structure in which all or half of the unstacked TMP molecules are replaced by DPE-I or DPE-II. Accordingly, one may understand the crystal structures of 3:2:2:1 MRE:TMP:ACR:DPE-I and MRE:TMP:ACR:DPE-II. In the 4:3:2:1 MRE:TMP:ACR:DPE-I cocrystal, only half the unstacked TMP molecules are replaced by DPE and the structure is of mechanistic relevance in the crystallization of the 3:2:2:1 solids (see S2 for details). The last quaternary is 3:2:2:1 MRE:TMP:ANT:DPE-II and it was obtained not through retrosynthesis but rather through a high throughput procedure. In this context, ANT occurs in a dead end ternary. In three of these four quaternaries (MRE:TMP:ACR:DPE-I, MRE:TMP:ACR:DPE-II, MRE:TMP:ANT:DPE-II) MRE molecules occur in ordered and disordered environments. When ordered, MRE forms O—H⋯N hydrogen bonds with DPE and TMP. When disordered, it lies on an inversion center and forms O—H⋯N hydrogen bonds with only TMP. These MRE sites represent a further inequivalence and the solid may be likened to **ABCD_1_D_2_** in Fig. 1 ▸. To summarize, the 10 quaternaries we have reported here may be divided into three groups: six of them are 2:1:1:1 **ABCD** solids and are synthetic dead ends; one is a 4:3:2:1 outlier; the other three are of the **ABCD_1_D_2_** type and could lend themselves to further development into a quintinary cocrystal.

**Figure 5:**
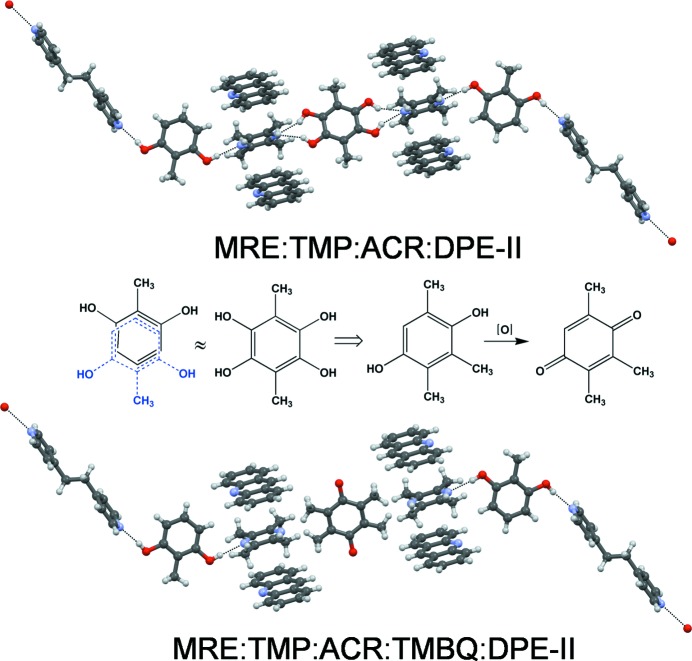
Retrosynthesis of quintinary solids. The MRE:TMP:ACR:DPE-II quaternary solid (top) has a disordered MRE that is replaced by the topologically similar TMBQ molecule in the crystal.

The synthetic strategy towards a five-component crystal uses the fact that the disordered MRE molecule in the ACR quaternaries (Fig. 2 ▸) is chemically and geometrically equivalent to a molecule of 1,2,4,5-tetrahydroxy-3,6-dimethylbenzene, and may therefore be replaced by it as a solid solution. This strategy has been used to make a ternary cocrystal from a binary (Bučar *et al.*, 2012 ▸). The tetrahydroxy molecule is, however, too unstable to isolate. A surrogate molecule, trimethylhydroquinone (TMHQ), based on OH/CH_3_ exchange, was identified. It was expected that TMHQ would replace the disordered MRE but not the ordered MRE. A mixture of ACR, DPE-II, MRE, TMHQ and TMP were taken together for crystallization in MeNO_2_, with the first four compounds in equimolar ratio and TMHQ in fivefold excess of the desired 100% occupancy in the disordered MRE site **D_2_**. A single solid was obtained in the form of yellow brown blocks; the quaternary is pale yellow to colorless (S3). A single crystal was selected and the cell parameters found to be identical to the quaternary. The same crystal was dissolved in MeOH and the GC–MS spectra traces recorded. The GC trace shows the clear presence of five components (S3). The crystal is therefore a five-component crystal. The MS identifies four of the compounds as MRE (*m*/*z* = 124), TMP (*m*/*z* = 136), DPE-II (*m*/*z* = 184) and ACR (*m*/*z* = 179), but the fifth compound is not TMHQ but rather its oxidation product 2,3,5-trimethyl-1,4-benzoquinone (TMBQ) with its characteristic molecular ion peak at *m*/*z* = 150. We thus identify (Fig. 5 ▸) the five-component solid (MRE)_3 − *x*_·TMP_2_·ACR_2_·DPE-II·TMBQ_*x*_ in which the fifth component, TMBQ, is not present in a stoichiometric amount.

Least-squares refinement of the X-ray data of the quintinary cocrystal shows the presence of TMBQ but the TMBQ:MRE ratio in the disordered inversion site cannot be estimated (S3). Considering that TMHQ is very prone to oxidation, and the crystal of the suspected quintinary is brown–yellow, it is concluded that TMHQ gets oxidized to TMBQ in solution and the latter enters the crystal in the disordered MRE site. TMBQ has the required topological similarities to occupy this site and has the shape–size mimicry with MRE that was anticipated for TMHQ. While TMBQ is bound in the site with C—H⋯N hydrogen bonds (S3), these are weaker than the O—H⋯N bonds formed by MRE. TMBQ cannot compete so well with the MRE for hydrogen bonding to TMP, but it still enters the crystal to some small extent (we estimate 5%) justifying the design strategy of using the lack of equivalence of the **D** site in the quaternary cocrystal to introduce the fifth component. There is a precedent for this kind of solid solution behavior in the system barbital–urea–acetamide (Thakur *et al.*, 2010 ▸). It is emphasized that the five-component solid obtained here is not, strictly speaking, a quintinary cocrystal, if by the latter term is meant a solid in which five different solid compounds are present in stoichiometric amounts. However, the present strategy outlines an approach to such cocrystals.

## 4. Conclusions   

Four- and higher-component molecular crystals can be designed with crystal engineering principles. In the 10 quaternary and one quintinary cocrystal reported here, the components are introduced into the solid using logic driven protocols and they occur in stoichiometric ratios, except for the fifth component TMBQ in the quintinary which enters the cocrystal in a solid solution manner. All the new solids we report contain the crucial components 2-methylresorcinol (MRE) and tetramethylpyrazine (TMP). In all cases there is an O—H⋯N hydrogen bond between these entities. Success rates in the crystallizations are quite high (around 75% and the unsuccessful 25% cases are mostly ones where a selected PAH molecule failed to give the desired product, see S4) and one may well ask why this is the case.

Some points need further discussion. The modularity of the entire sequence would seem to hint that synthons present in the crystals are also present in the crystallizing solution. Once the binary to ternary progression was made, it was possible to think of the ternary to quaternary progression in the same terms: the key elements of the ternary structures are likely to be present in solution, and the fourth component is seemingly ‘added’ to give the quaternary. One of the reasons for the success rate of this strategy could be that none of the components contain very good hydrogen bond donor or acceptor groups. The strongest hydrogen bond in the system is the O—H_phenol_⋯N_pyridine_ interaction. There are no acids, amides and other similar compounds in any of these new cocrystals. Good donors and acceptors lead necessarily to strong hydrogen bonds and these lead to stable lower component cocrystals (binary, ternary) that act as synthetic terminators or dead ends. The essence of making a quaternary (or higher) cocrystal seems to be a selection of molecules, all of which associate with comparable intermolecular interactions. Perhaps it is also this feature that allows for the breakdown of structural equivalence (**B** → **B_1_, B_2_**; **C** → **C_1_, C_2_**; **D** → **D_1_, D_2_**) throughout the reaction cascade, and which has been used to make ternaries from binaries, quaternaries from ternaries and so on. Also of interest is the idea of sites that are ‘potentially’ inequivalent. In the MRE:TMP binary in Fig. 2 ▸, all TMP molecules are identical. However, C—H⋯π stacking with a PAH causes an inequivalence (**C** → **C_1_, C_2_**) in cases where the PAH is larger or more polar (ACR, PER, PHE, TOL) and where perhaps stacking at the second site is hindered because of stereoelectronic factors. Smaller and/or less polar PAH molecules (ANT, HMB, PYR) stack at both sites leading to dead end ternaries.

Crystallization proceeds from solution and this modularity is very strong evidence for the persistence of small and large supramolecular synthons in solution (Mukherjee *et al.*, 2014 ▸). The nucleation of a higher component crystal may be visualized as occurring *via* the attachment of the *n*th component to an (*n* − 1) cluster in solution. This is the crux of synthon theory (Desiraju, 1995 ▸). The design of each new crystal is not an *ab initio* exercise (Dunitz, 2015 ▸; Thakur *et al.*, 2015 ▸; Lecomte *et al.*, 2015 ▸). Smaller and larger synthons and clusters are sufficiently stable kinetically so that crystal build-up can be analyzed retrosynthetically.

## Supplementary Material

Crystal structure: contains datablock(s) global, MRE_TMP_22BP, MRE_TMP_22TP, MRE_TMP_ACR, MRE_TMP_ANT, MRE_TMP_DP, MRE_TMP_DPE-I_22BP, MRE_TMP_DPE-I_22TP, Form_I_MRE_TMP_DPE-I_ACR, Form_II_MRE_TMP_DPE-I_ACR, MRE_TMP_DPE-I_DP, MRE_TMP_DPE-II_22BP, MRE_TMP_DPE-II_22TP, MRE_TMP_DPE-II_ACR, MRE_TMP_DPE-II_ACR_TMBQ, MRE_TMP_DPE-II_ANT, MRE_TMP_DPE-II_DP, MRE_TMP_HMB, MRE_TMP_PER, MRE_TMP_PHE, MRE_TMP_PYR, MRE_TMP_TOL. DOI: 10.1107/S2052252515023945/hi5640sup1.cif

Supporting information. DOI: 10.1107/S2052252515023945/hi5640sup2.pdf

GC-MS Dataset. DOI: 10.1107/S2052252515023945/hi5640sup3.zip

Refinement Models for Quintinary Solid. DOI: 10.1107/S2052252515023945/hi5640sup4.zip

CIF files. DOI: 10.1107/S2052252515023945/hi5640sup6.zip

CCDC references: 1427989, 1428000, 1428004, 1428005, 1428006, 1427991, 1427992, 1427998, 1427999, 1427995, 1427993, 1427994, 1428001, 1428002, 1427997, 1427996, 1428007, 1428008, 1428009, 1427990, 1428003
