# Supplementary material for: Four- and five-component molecular solids: crystal engineering strategies based on structural inequivalence
Source: IUCrJ. 2016 Jan 5;3(Pt 2):96–101. doi: 10.1107/S2052252515023945 (PMC4775157; doi:10.1107/S2052252515023945)
Supplement: Supplementary file 2 [file m-03-00096-sup2.pdf]

IUCrJ

Volume 3 (2016)

Supporting information for article:

Four- and five-component molecular solids: crystal engineering strategies based on structural inequivalence

Niyaz A. Mir, Ritesh Dubey and Gautam R. Desiraju

Contents

S1: Crystallization details

S2: Mechanistic relevance to the crystallization. From ternary to quaternary

S3: Evidence to show the presence of the fifth component (TMBQ) in the proposed quintinary cocrystal MRE.TMP.ACR.TMBQ.DPE-II

S4: Failed experimental efforts for the crystal synthesis of quaternary and quintinary solids

S5: Crystallographic tables

S1. Crystallization details

a) *2-Methylresorcinol.Tetramethylpyrazine.*2,2'-*Bipyridine* **(1)***:* An equimolar mixture of 2-methylresorcinol, tetramethylpyrazine and 2,2'-bipyridine along with 2-3 drops of THF was ground in a mortar with a pestle for ten minutes. Colorless block shaped diffraction quality crystals were obtained from nitromethane after 3-4 days (mp=149.5 ^{o}C).

b) *2-Methylresorcinol.Tetramethylpyrazine.*2,2'-*Bisthiophene* **(2)***:* An equimolar mixture of 2-methylresorcinol, tetramethylpyrazine and 2,2'-*bis*thiophene along with 2-3 drops of THF was ground in a mortar with a pestle for ten minutes. Colorless block shaped diffraction quality crystals were obtained after 3-4 days from nitromethane at 5°C (mp=155.7 ^{o}C).

c) *2-Methylresorcinol.Tetramethylpyrazine.Tolan* **(3)***:* An equimolar mixture of 2-methylresorcinol, tetramethylpyrazine and tolan was ground in a mortar with a pestle for ten minutes with 2-3 drops of THF. Colorless plate like diffraction quality crystals were obtained after 4 days from nitromethane (mp=152.8 ^{o}C).

d) *2-Methylresorcinol.Tetramethylpyrazine.Acridine* **(4)**: Liquid assisted grinding was performed on a 1:1:1 mixture of the precursor compounds with tetrahydrofuran (THF) as a solvent. Light green colored diffraction quality crystals were obtained in 2-3 days from nitromethane (mp=158 ^{o}C).

e) *2-Methylresorcinol.Tetramethylpyrazine.Anthracene* **(5)***:* A 1:1:1 mixture of 2-methylresorcinol, tetramethylpyrazine and anthracene along with 2-3 drops of THF was ground in a mortar with a pestle for ten minutes. Colorless block shaped diffraction quality crystals were obtained after 5 days from nitromethane (mp=191.9 ^{o}C).

f) *2-Methylresorcinol.Tetramethylpyrazine.Biphenyl* **(6)***:* An equimolar mixture of 2-methylresorcinol, tetramethylpyrazine and biphenyl along with 2-3 drops of THF was ground in a mortar with a pestle for ten minutes. Colorless block shaped diffraction quality crystals were obtained after 3-4 days from nitromethane (mp=145.5 ^{o}C).

g) *2-Methylresorcinol.Tetramethylpyrazine.Hexamethylbenzene* **(7)***:* A 1:1:1 mixture of 2-methylresorcinol, tetramethylpyrazine and hexamethylbenzene along with 2-3 drops of THF was ground in a mortar with a pestle for ten minutes. Colorless block shaped diffraction quality crystals were obtained after 5 days from nitromethane (mp=142.5 ^{o}C).

h) *2-Methylresorcinol.Tetramethylpyrazine.Perylene* **(8)***:* Liquid assisted grinding was performed on a 1:1:1 mixture of the precursor compounds with THF as a solvent. Yellow colored block shaped diffraction quality crystals were obtained in 2-3 days from benzene (mp=247 °C).

i) *2-Methylresorcinol.Tetramethylpyrazine.Phenazine* **(9)**: An equimolar mixture of 2-methylresorcinol, tetramethylpyrazine and phenazine along with 2-3 drops of THF was ground in a mortar with a pestle for ten minutes. Green colored diffraction quality crystals were obtained after 5 days from nitromethane (mp=160.5 °C).

j) *2-Methylresorcinol.Tetramethylpyrazine.Pyrene* **(10)***:* An equimolar mixture of 2-methylresorcinol, tetramethylpyrazine and pyrene along with 2-3 drops of THF was ground in a mortar with a pestle for ten minutes. Colorless block shaped diffraction quality crystals were obtained after 3-4 days from nitromethane 5°C (mp=173.5 °C).

k) *2-Methylresorcinol.Tetramethylpyrazine.*2,2'-*Bipyridine.*1,2-*Bis*(4-*pyridyl*)*ethene* **(11)***:* A 3:2:2:2 mixture of 2-methylresorcinol, tetramethylpyrazine, 2,2'-bipyridine and 1,2-*bis*(4-pyridyl)ethene along with 2-3 drops of THF was ground in a mortar with a pestle for ten minutes. Colorless block shaped diffraction quality crystals were obtained after 3 days from nitromethane (mp=130.0 °C).

l) *2-Methylresorcinol.Tetramethylpyrazine.*2,2'-*Bisthiophene:* 1,2-*bis*(4-*pyridyl*)*ethene* **(12)***:* A 3:2:2:2 mixture of 2-methylresorcinol, tetramethylpyrazine, 2,2'-*bis*thiophene and 1,2-*bis*(4-pyridyl)ethene along with 2-3 drops of THF was ground in a mortar with a pestle for ten minutes. Light brown block shaped diffraction quality crystals were obtained after 5 days from nitromethane (mp=154.4 °C).

m) *2-Methylresorcinol.Tetramethylpyrazine.*2,2'-*bipyridine.1,2-Bis*(4-*pyridyl*)*ethane* **(13)***:* A 3:2:2:2 mixture of 2-methylresorcinol, tetramethylpyrazine, 2,2'-bipyridine and 1,2-*bis*(4-pyridyl)ethane along with 2-3 drops of THF was ground in a mortar with a pestle for ten minutes. Colorless block shaped diffraction quality crystals were obtained after 3 days from nitromethane (mp=163.0 °C).

n) *2-Methylresorcinol.Tetramethylpyrazine.*2,2'-*Bisthiophene.*1,2-*Bis*(4-*pyridyl*)*ethane* **(14)***:* A 3:2:2:2 mixture of 2-methylresorcinol, Tetramethylpyrazine, 2,2'-*bis*thiophene and 1,2-*bis*(4-pyridyl)ethane along with 2-3 drops of THF was ground in a mortar with a pestle

for ten minutes. Colorless block shaped diffraction quality crystals were obtained after 5 days from nitromethane (mp=143.2 ^{o}C).

o) *2-Methylresorcinol.Tetramethylpyrazine.B*iphenyl.*1,2-Bis*(4-*pyridyl*)*ethene* **(15)***:* A 3:2:2:2 mixture of 2-methylresorcinol, tetramethylpyrazine, biphenyl and 1,2-*bis*(4-pyridyl)ethene along with 2-3 drops of THF was ground in a mortar with a pestle for ten minutes. Light brown block shaped diffraction quality crystals were obtained after 5 days from a (1:1) mixture of nitromethane at 5°C (mp=137.6 ^{o}C).

p) *2-Methylresorcinol.Tetramethylpyrazine.Biphenyl.1,2-Bis*(4-*pyridyl*)*ethane* **(16)***:* A 3:2:2:2 mixture of 2-methylresorcinol, tetramethylpyrazine, biphenyl and 1,2-*bis*(4-pyridyl)ethane along with 2-3 drops of THF was ground in a mortar with a pestle for ten minutes. Colorless block shaped diffraction quality crystals were obtained after 3 days from a (1:1) mixture of nitromethane and benzene (mp=140.0 ^{o}C).

q) *2-Methylresorcinol.Tetramethylpyrazine.Anthracene.*1,2-*Bis*(4-*pyridyl*)*ethane* **(17)***:* A 3:2:2:2 mixture of 2-methylresorcinol, tetramethylpyrazine, anthracene and 1,2-*bis*(4-pyridyl)ethane along with 2-3 drops of THF was ground in a mortar with a pestle for ten minutes. Light green colored block shaped diffraction quality crystals were obtained after 5 days from nitromethane at 5°C (mp=181.7 ^{o}C).

r) *2-Methylresorcinol.Tetramethylpyrazine.Acridine.*1,*2-Bis*(4-*pyridyl*)*ethene* **(18)***:* A 1:2:1:1 mixture of 2-methylresorcinol, tetramethylpyrazine, acridine and 1,2-*bis*(4-pyridyl)ethene along with 2-3 drops of THF was ground in a mortar with a pestle for ten minutes. Light green colored block shaped diffraction quality crystals were obtained after 5 days from nitromethane (mp=172.5 ^{o}C).

s) *2-Methylresorcinol.Tetramethylpyrazine.Acridine.1,2-Bis*(4-*pyridyl*)*ethene* **(19)***:* A 3:2:2:2 mixture of 2-methylresorcinol, tetramethylpyrazine, acridine and 1,2-*bis*(4-pyridyl)ethene along with 2-3 drops of THF was ground in a mortar with a pestle for ten minutes. Pale yellow colored block shaped diffraction quality crystals were obtained after 5 days from nitromethane (mp=190.5 ^{o}C).

t) *2-Methylresorcinol.Tetramethylpyrazine.Acridine*.1,2-*Bis*(4-*pyridyl*)*ethane* **(20)***:* A 3:2:2:2 mixture of 2-methylresorcinol, tetramethylpyrazine, acridine and 1,2-*bis*(4-pyridyl)ethane along with 2-3 drops of THF was ground in a mortar with a pestle for ten

minutes. Pale yellow colored block shaped diffraction quality crystals were obtained after 5 days from nitromethane at 5°C (mp=151.5 ^{o}C).

u) *2-Methylresorcinol*.*Tetramethylpyrazine*.*Acridine*:*1*,*2-Bis*(4-*pyridyl*)*ethane*.*2*,*3*,*5-Trimethylbenzoquinone* **(21)***:* A 2:2:2:2:3 mixture of 2-methylresorcinol, tetramethylpyrazine, acridine, 1,2-*bis*(4-pyridyl)ethane and 2,3,5-trimethylhydroquinone was ground in a mortar with a pestle along with 2-3 drops of THF. Brown colored block shaped diffraction quality crystals were obtained after 5 days from nitromethane (mp=153.2 ^{o}C).

S2. Mechanistic relevance to the crystallization. From ternary to quaternary

The isolation of quaternary cocrystals may be mechanistically understood by the following figure where 1:2:1 MRE.TMP.ACR is a starting point of the type **ABC₁C₂**. After replacing one or both unstacked TMP molecules, the ternary cocrystal will eventually lead to the isolation of 4:3:2:1 MRE.TMP.ACR.DPE-I or 3:2:2:1 MRE.TMP.ACR.DPE-I quaternary cocrystals, respectively. This could be the actual sequence of events in all cases.

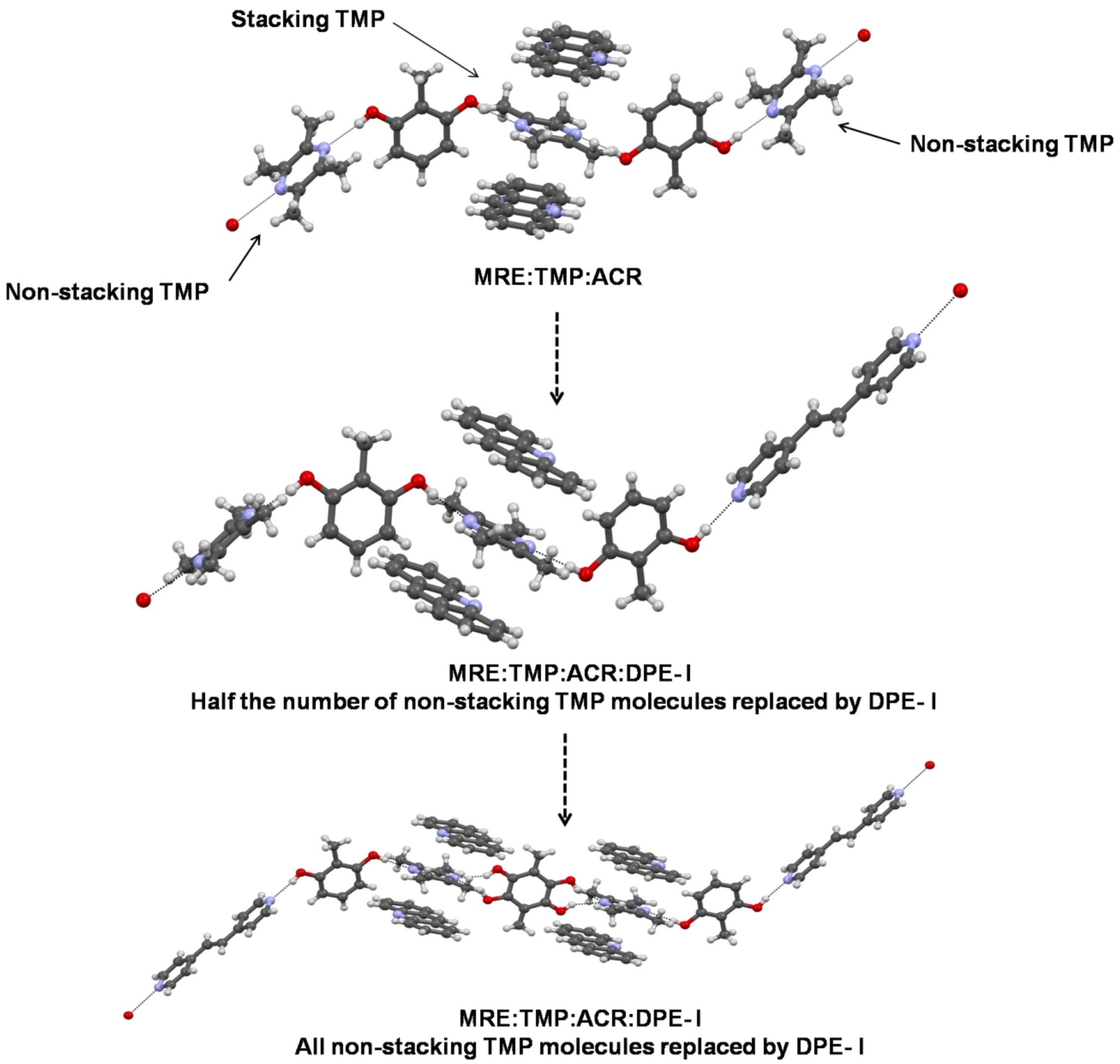

S3. Evidence to show the presence of the fifth component (TMBQ) in the proposed quintinary cocrystal MRE.TMP.ACR.TMBQ.DPE-II

The reference compound is the quaternary cocrystal MRE.TMP.ACR.DPE-II, the structure of which is shown below:

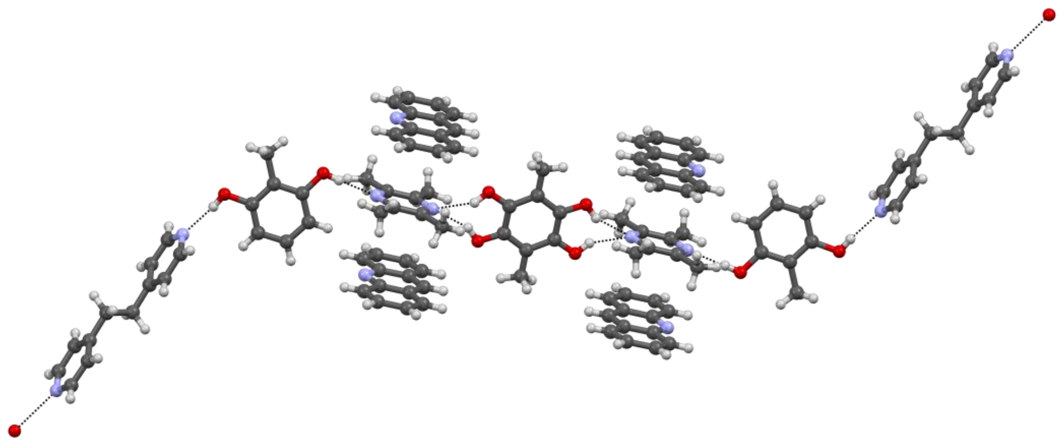

It is noted that the central MRE molecule is disordered about an inversion center. The two alternative positions of the MRE molecules are shown below, from which it is seen that in both cases O–H…N hydrogen bonding is possible.

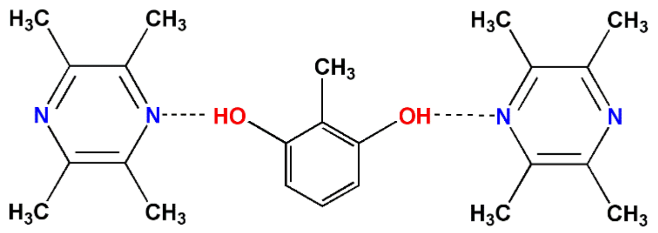

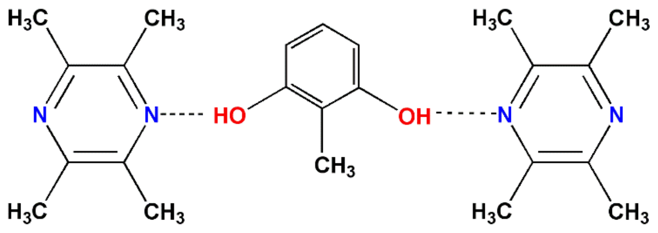

The crystal of the quaternary is pale yellow to colorless.

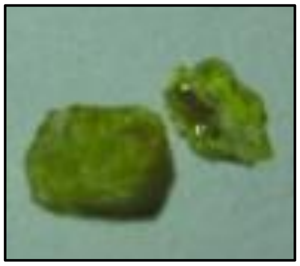

The design strategy involves cocrystallization of the four components above with trimethylhydroquinone (TMHQ). TMHQ is supposed to enter the crystal in a solid solution manner and in other words act as a shape and interaction mimic of the disordered MRE. The two expected alternative disordered positions of the TMHQ molecule are shown below:

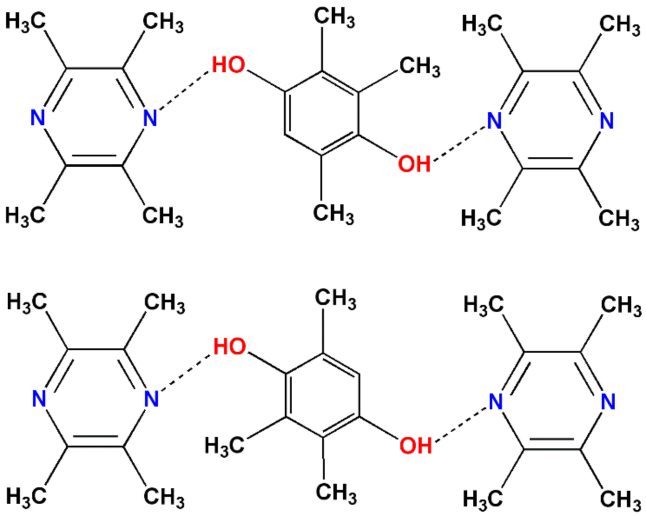

It is seen that the TMHQ molecule is O–H…N hydrogen bonded in each of the expected disordered positions.

The new crystal is brown yellow

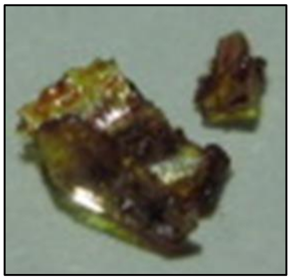

A single crystal is taken and the cell parameters are determined. The cell parameters are the same as that of the quaternary. This one crystal is dissolved in CH_3OH and the GC-MS spectra are recorded. The GC trace shows the clear presence of five components. The crystal is therefore a quintinary solid. The MS identifies the five compounds as MRE (m/z=124), TMP (m/z= 136), DPE-II (m/z=184), ACR (m/z=179) but the fifth compound is not TMHQ but rather its oxidation product 2,3,5-trimethyl-1,4-benzoquinone (TMBQ) with its characteristic molecular ion peak at m/z=150.

Considering that TMHQ is very prone to oxidation, and the crystal of the suspected quintinary is brown-yellow, it is concluded that TMHQ gets oxidized in solution and the TMBQ that is produced enters the crystal in the disordered MRE site. TMBQ has the required topological similarities to enter the disordered MRE site and facilitates the shape-size mimicry that was anticipated for TMHQ. The two orientations of TMBQ are given below:

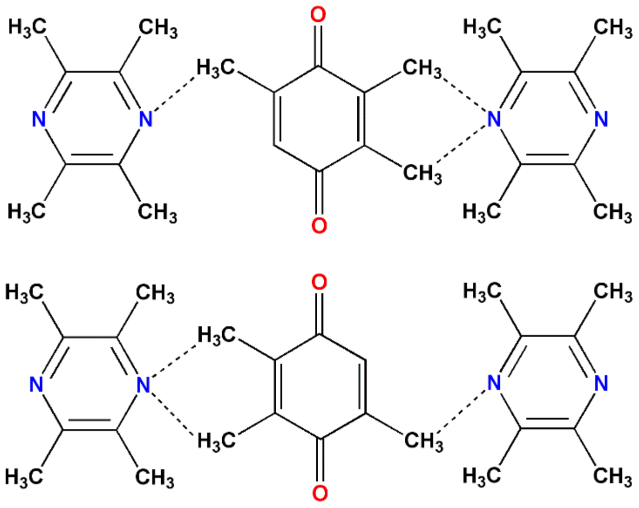

It is noted that TMBQ is bound in the site with C–H…N hydrogen bonds rather than the expected O–H…N bonds with MRE. This is a weaker interaction and we conclude that the amount of TMBQ in the site will be much less than MRE. It cannot compete so well with the MRE but it still enters the crystal to some small extent justifying the design strategy of exploiting the lack of degeneracy of the **D** site in the quaternary cocrystal. There is a precedent for this kind of behavior (strong versus weak hydrogen bonding) and we have observed similar solid solutions in the system barbital–urea–acetamide (Ref. 29).It is not clear why TMHQ does not enter the crystal (it is present in large excess in the mother liquor). Perhaps, it is a steric factor that allows for the entry of the TMBQ molecule and not TMHQ.

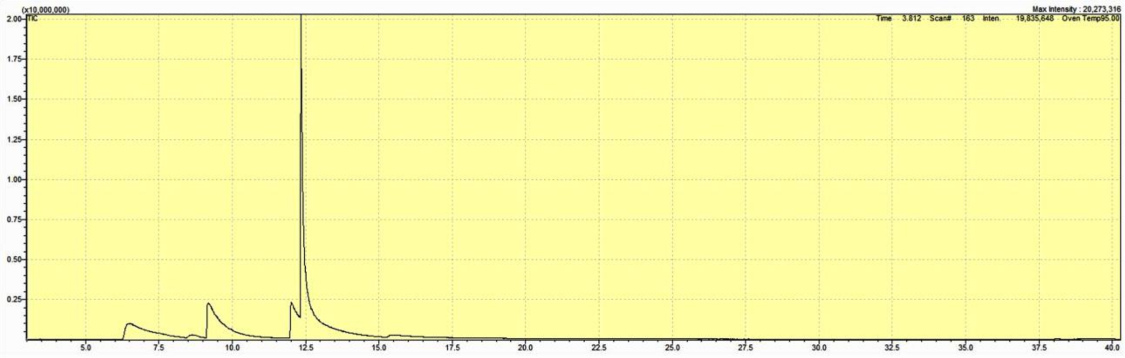

Peak Report TIC

Peak#	R.Time	I.Time	F.Time	Area	Area%	Height	Height%	A/H	Mark	Name
1	6.490	6.200	8.140	47982597	20.39	973480	4.11	49.29	MI	
2	8.637	8.400	9.020	3357307	1.43	175812	0.74	19.10	MI	
3	9.190	9.045	10.525	64484714	27.40	2161161	9.13	29.84	MI	
4	12.017	11.820	12.250	11980744	5.09	1585720	6.70	7.56	MI	
5	12.344	12.225	13.800	107538886	45.69	18769446	79.31	5.73	MI	
				235344248	100.00	23665619	100.00			

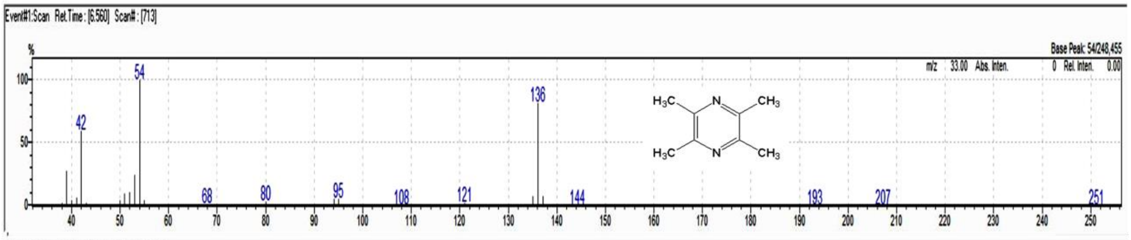

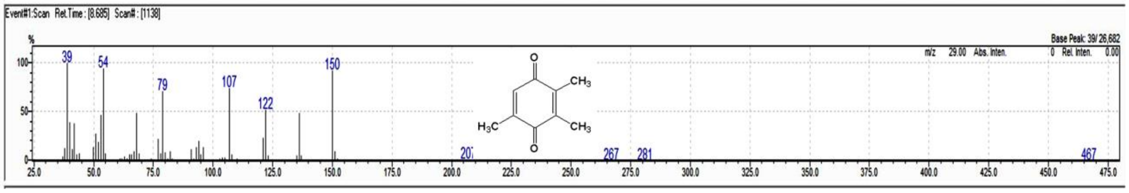

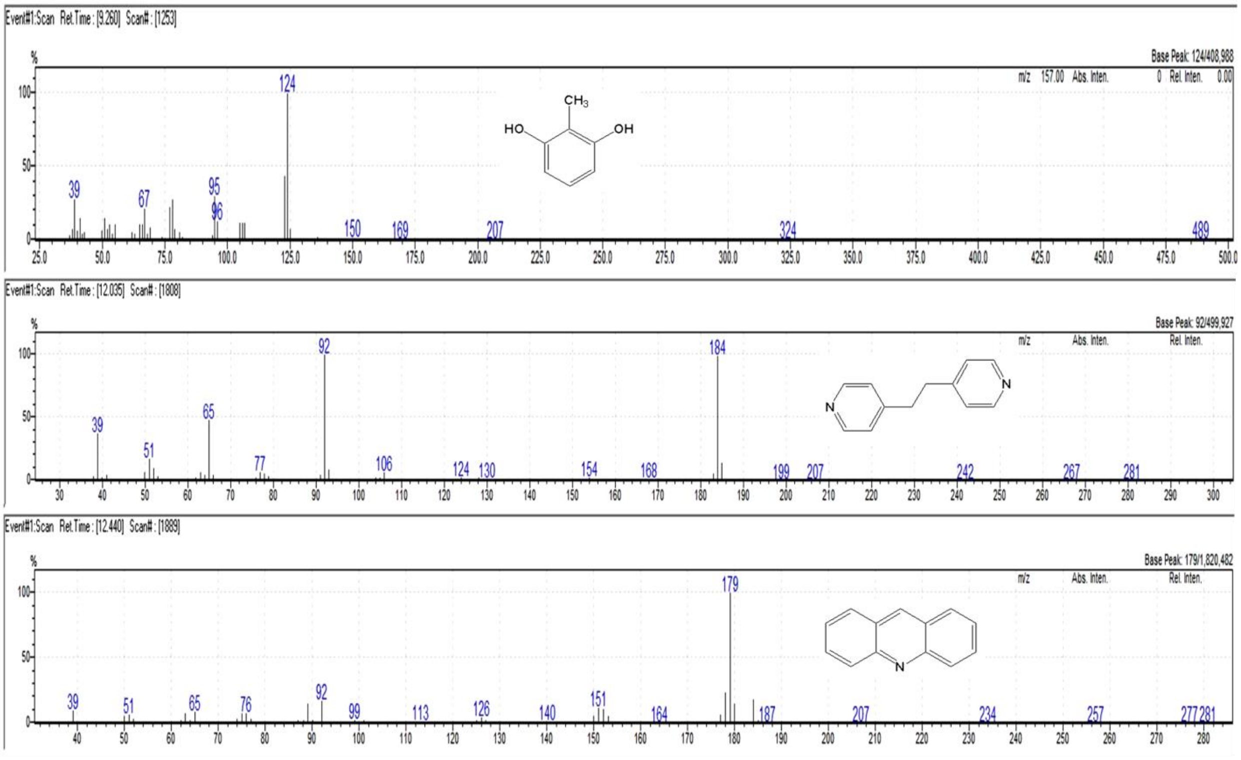

S3.1. Refinement of MRE and TMBQ molecules in the disordered positions of the proposed quintinary solid

In the general case, both MRE and TMBQ molecules are present at the inversion site. Least squares refinement of the data is on a model that has a six membered benzene ring with electron density corresponding to C/O atoms on all six positions of the ring.

Summary: Least squares refinement of the data on the brown crystal shows the presence of TMBQ in the crystal but it is unable to determine the ratio of TMBQ:MRE in the disordered site.

Details are given below:

Because of the inversion center disorder there are two C-atom positions and four O-atom positions for the MRE, each of which has site occupancy factor (SOF) of 0.5, if the MRE molecule has 100% occupancy at the site. If the disordered TMBQ molecule is present to the extent of 100% on

this site, the expected SOF will be 1.0 for the O-atom and 0.75 for each of the four C-atoms. From the observed numbers in the refinements, it seems likely that both MRE and TMBQ are present at this disordered site in some statistical manner.

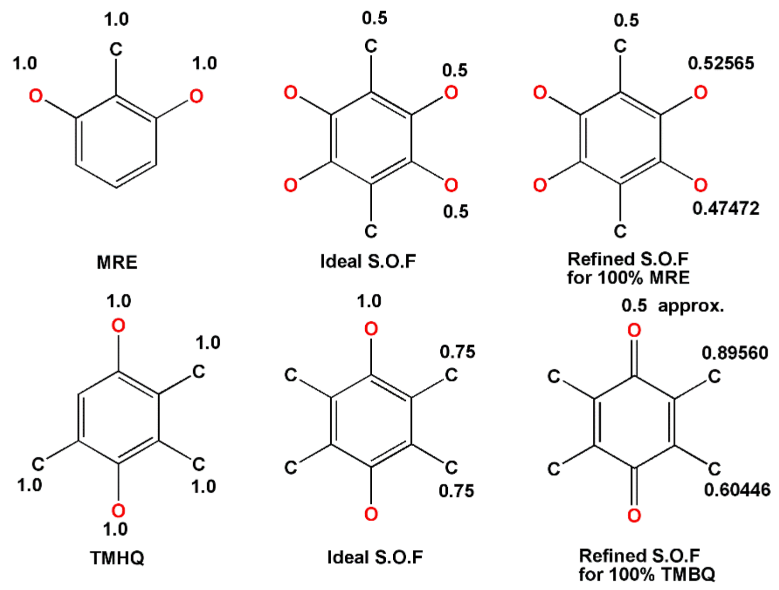

The above scheme shows the SOF of the C/O atoms assuming 100% MRE occupancy and 100% TMBQ occupancy. Neither set of numbers in the actual refinements correspond exactly to the ideal numbers for 100% occupancy for each of the components MRE and TMBQ. *However, both refinements are stable and no atom becomes non-positive definite*. The electron density maps below convey this:

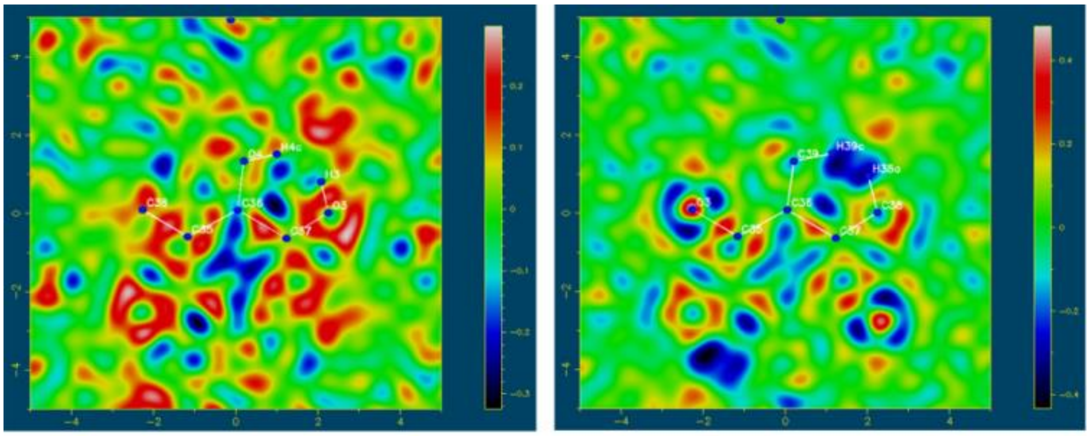

Electron density maps of proposed brown (quintinary) crystals with alternative refinement models. Left: model with 100% MRE; Right: model with 100% TMBQ.

Since both models are reasonable (MRE 100% and TMBQ 100%) to some degree, we conclude that the site contains both molecules.

The final ORTEP figure is shown below:

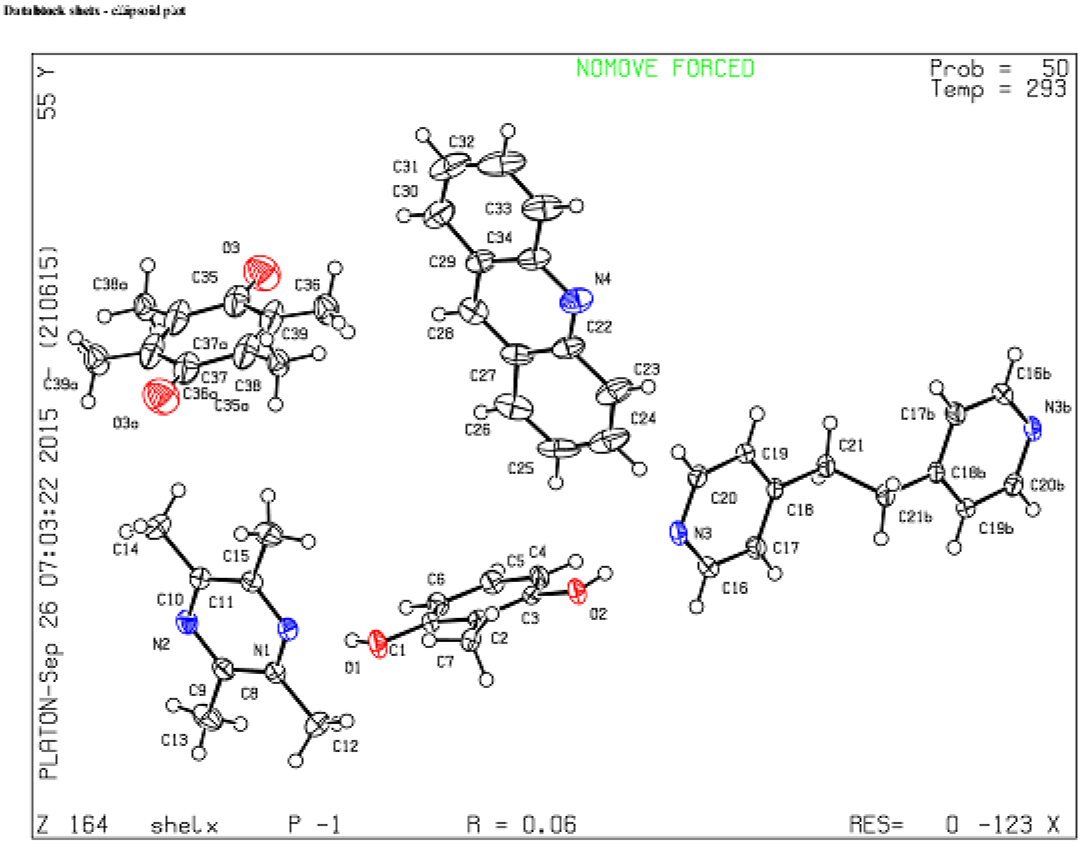

S3.2. Confirmation of the presence of TMBQ in the disordered site

Similar refinements were carried out (100% MRE and 100% TMBQ in the central inversion site) for the data of the yellow (quaternary) crystal. In this case, it was found that the refinement with 100% MRE proceeded satisfactorily (as expected). Refinement with 100% TMBQ (which we know is patently incorrect) did not proceed satisfactorily. *The refinement was unstable and several H-atoms became non-positive definite.* This indicates that the data on both the yellow and

brown crystals is good enough to show that some TMBQ is present in the brown crystal. The data is at a better level than where either or both MRE and TMBQ may be placed at the inversion site in either data set and refined normally. INS files for the datasets (in text format) are provided for the reviewers.

We estimate that the amount of TMBQ at the disordered site is around 5% (MRE is 95%) because there is no evidence of any C=O stretching absorption in the IR spectrum of the quintinary cocrystal.

S4. Failed experimental efforts for the crystal synthesis of quaternary and quintinary solids

	**Compounds**	**Ratio**	**Result**
1	MRE:TMP:PYR:DPE-II	1:2:1:1/3:2:2:2	Ternary (MRE:TMP:PYR)
2	MRE:TMP:PYR:DPE-I	1:2:1:1/3:2:2:2	Binary(MRE:DPE-I)
3	MRE:TMP:PER:DPE-II	1:2:1:1/3:2:2:2	Binary(MRE:DPE-II)<br>Ternary (MRE:TMP:PER)
4	MRE:TMP:HMB:DPE-II	1:2:1:1/3:2:2:2	Binary(MRE:DPE-II)<br>Ternary (MRE:TMP:HMB)
5	MRE:TMP:TOL:DPE-II	1:2:1:1/3:2:2:2	Binary(MRE:DPE-II)<br>Ternary (MRE:TMP:TOL)
6	MRE:TMP:ANT:DPE-I	1:2:1:1/3:2:2:2	Ternary (MRE:TMP:ANT)
7	MRE:TMP:PHE:DPE-II	1:2:1:1/3:2:2:2	Ternary (MRE:TMP:PHE)
8	MRE:TMP:DPE-I:ACR:44BPY	2:2:1:2:1	Quaternary(MRE:TMP:DPE-I:ACR)

*Solvents and mixture of solvents used in the high-throughput crystallization experiments–

-- Nitromethane, acetonitrile, tetrahydrofuran, methanol, ethyl acetate and ethanol

-- 1:1 nitromethane:benzene and 1:1 acetonitrile:benzene.

S5. Crystallographic tables

Compound numbers are given in the section on crystallization details

Compound	1	2	3	4	5	6
CCDC No.	1427989	1428000	1428003	1428004	1428005	1428006
Mol. Formula	$C_{32}H_{36}N_4O_4$	$C_{30}H_{34}N_2O_4S_2$	$C_{44}H_{50}N_4O_4$	$C_{43}H_{49}N_5O_4$	$C_{29}H_{30}N_2O_2$	$C_{34}H_{38}N_2O_4$
Formula Weight	540.65	550.73	698.88	699.87	438.55	538.66
Crystal System	Monoclinic	Monoclinic	Triclinic	Triclinic	Monoclinic	Monoclinic
Space Group	$P2_1$/n	$P2_1$/n	P 1	P 1	$P2_1$/c	$P2_1$/n
a (Å)	7.8566(12)	7.7971(14)	7.195(7)	7.409(10)	7.5571(7)	8.292(6)
b (Å)	21.336(4)	22.217(5)	8.865(8)	8.781(11)	24.125(2)	20.615(15)
c (Å)	8.3872(17)	7.9179(15)	15.981(15)	15.360(19)	12.9363(13)	8.470(6)
α (°)	90	90	80.52(3)	81.63(3)	90	90
β (°)	90.911(11)	91.479(15)	84.20(3)	86.57(3)	92.034(4)	92.435(7)
γ (°)	90	90	79.19(2)	78.64(2)	90	90
V ($Å^3$)	1405.8(4)	1371.2(5)	985.0(16)	969.0(2)	2357.0(4)	1446.6(18)
ρ_{calc} (g/cm^3)	1.277	1.334	1.178	1.199	1.236	1.237
F(000)	576	584	374	374	936	576
μ. (mm^{-1})	0.085	0.233	0.076	0.078	0.077	0.081
T (K)	150(2)	150(2)	150(2)	150(2)	150(2)	150(2)
λ (Å)	0.71073	0.71073	0.71073	0.71073	0.71073	0.71073
Total Reflns.	14373	13884	10347	10247	24797	14988
Unique Reflns.	3224	3151	4481	4377	5411	3328
Comple. (%)	99.7	99.7	99.7	99.7	99.9	99.6
R_{int}	0.092	0.080	0.133	0.163	0.116	0.076
R_1 (F^2)	0.0561	0.0532	0.0629	0.0804	0.0677	0.0652
wR_2(F^2)	0.1744	0.1676	0.1984	0.2530	0.2103	0.2203
GooF	1.09	1.01	0.99	0.93	1.08	0.99
2θ	54	54	54	54	54	54

Compound	7	8	9	10	11	12
CCDC No.	1428007	1428008	1428009	1427990	1427991	1427992
Mol. Formula	$C_{27}H_{38}N_2O_2$	$C_{50}H_{52}N_4O_4$	$C_{42}H_{48}N_6O_4$	$C_{31}H_{30}N_2O_2$	$C_{44}H_{46}N_6O_4$	$C_{42}H_{44}N_4\ S_2O_4$
Formula Weight	422.59	772.96	700.86	462.57	722.87	732.95
Crystal System	Monoclinic	Triclinic	Triclinic	Monoclinic	Triclinic	Triclinic
Space Group	$P2_1/c$	P 1	P 1	$P2_1/c$	P 1	P 1
a (Å)	8.356(4)	7.5460(9)	7.324(7)	8.0059(6)	7.485(6)	7.492(5)

**b (Å)**	24.894(12)	9.4647(16)	8.723(8)	24.903(2)	8.797(7)	9.069(6)
**c (Å)**	11.744(6)	14.8318(19)	15.107(14)	12.0892(9)	14.171(12)	13.643(10)
**α (°)**	90	85.58(2)	82.27(3)	90	96.618(13)	98.069(13)
**β (°)**	96.324(6)	77.14(2)	88.73(3)	91.065(3)	93.306(12)	92.516(13)
**γ (°)**	90	86.77(3)	78.76(2)	90	91.826(14)	91.428(8)
**V ($Å^3$)**	2428.0(2)	1028.8(3)	938.0(15)	2409.8(3)	924.6(13)	916.5(11)
**ρ_{calc} (g/cm^3)**	1.156	1.248	1.241	1.275	1.298	1.328
**F(000)**	920	412	374	984	384	388
**μ. (mm^{-1})**	0.072	0.079	0.081	0.080	0.085	0.195
**T (K)**	150(2)	150(2)	150(2)	150(2)	150(2)	150(2)
**λ (Å)**	0.71073	0.71073	0.71073	0.71073	0.71073	0.71073
**Total Reflns.**	25296	11018	10049	24699	9807	9797
**Unique Reflns.**	5587	4739	4317	5522	4241	4205
**Comple. (%)**	99.7	99.3	99.4	99.8	99.0	99.4
**R_{int}**	0.075	0.077	0.069	0.103	0.069	0.074
**R_1 (F^2)**	0.0679	0.0623	0.0636	0.0638	0.0486	0.0479
**wR_2(F^2)**	0.2239	0.2124	0.2128	0.1956	0.1632	0.1480
**GooF**	1.049	1.10	1.08	1.07	1.09	1.01
**2θ**	54	54	54	54	54	54

Compound	13	14	15	16	17	18
**CCDC No.**	1427993	1427994	1427995	1427996	1427997	1427998
**Mol. Formula**	$C_{44}H_{48}N_6O_4$	$C_{42}H_{46}N_4O_2S_2$	$C_{46}H_{48}N_4O_4$	$C_{46}H_{50}N_4O_4$	$C_{77}H_{77}N_6O_6$	$C_{90}H_{96}N_{10}O_8$
**Formula Weight**	724.88	734.97	720.88	722.90	1182.40	1445.77
**Crystal System**	Triclinic	Triclinic	Triclinic	Triclinic	Triclinic	Triclinic
**Space Group**	*P*1	*P*1	*P*1	*P*1	*P*1	*P*1
**a (Å)**	7.4957(13)	7.531(9)	7.528(11)	7.5407(12)	7.4914(3)	7.3795(10)
**b (Å)**	8.7882(17)	9.026(12)	8.948(14)	8.9198(13)	8.8265(4)	8.8871(13)
**c (Å)**	14.308(3)	13.941(17)	14.11(2)	14.284(3)	23.583(1)	29.533(5)
**α (°)**	97.049(11)	98.18(2)	96.219(17)	96.506(7)	92.722(1)	89.495(17)
**β (°)**	94.334(13)	92.03(3)	94.61(2)	96.093(10)	92.976(1)	84.054(16)
**γ (°)**	91.781(10)	91.235(12)	90.598(13)	90.634(10)	91.626(1)	85.281(10)
**V ($Å^3$)**	932.0(3)	937.0(2)	942.0(2)	948.9(3)	1554.77(11)	1919.9(5)
**ρ_{calc} (g/cm^3)**	1.291	1.303	1.271	1.265	1.263	1.250
**F(000)**	386	390	384	386	629	770
**μ. (mm^{-1})**	0.084	0.190	0.081	0.081	0.080	0.081
**T (K)**	150(2)	150(2)	150(2)	150(2)	150(2)	150(2)

**λ (Å)**	0.71073	0.71073	0.71073	0.71073	0.71073	0.71073
**Total Reflns.**	10002	8797	8263	10144	36300	19146
**Unique Reflns.**	4287	3673	3646	4338	6096	8793
**Comple. (%)**	99.5	99.6	98.4	99.2	99.8	98.6
**R_{int}**	0.108	0.061	0.066	0.093	0.080	0.091
**R_1 (F^2)**	0.0565	0.0823	0.0511	0.0541	0.0545	0.0688
**wR_2(F^2)**	0.1804	0.2817	0.1668	0.1748	0.1641	0.2293
**GooF**	1.05	1.16	1.06	0.89	1.06	0.109
**2θ**	54	52	52	54	52	54

**Compound**	**19**	**20**	**21**
**CCDC No.**	1427999	1428001	1428002
**Molecular Formula**	$C_{75}H_{73}N_8O_6$	$C_{75}H_{75}N_8O_6$	$C_{77}H_{80}N_8O_5$
**Formula Weight**	1182.41	1184.43	1197.49
**Crystal System**	Triclinic	Triclinic	Triclinic
**Space Group**	*P*1	*P*1	*P*1
**a (Å)**	7.478(12)	7.4611(3)	7.4709(3)
**b (Å)**	8.739(14)	8.7285(4)	8.7326(3)
**c (Å)**	23.37(3)	23.6219(10)	23.6381(9)
**α (°)**	92.36(3)	92.850(1)	92.857(1)
**β (°)**	90.29(3)	91.347(1)	91.317(1)
**γ (°)**	93.131(19)	93.317(1)	93.293(1)
**V ($Å^3$)**	1524(4)	1533.34(11)	1537.18(10)
**ρ_{calc} (g/cm^3)**	1.288	1.283	1.294
**F(000)**	627	629	638
**μ. (mm^{-1})**	0.083	0.082	0.082
**T (K)**	150(2)	150(2)	150(2)
**λ (Å)**	0.71073	0.71073	0.71073
**Total Reflns.**	13754	39279	80029
**Unique Reflns.**	7022	7056	7136
**Completeness (%)**	96.1	99.6	99.7
**R_{int}**	0.113	0.023	0.048
**R_1 (F^2)**	0.0948	0.0553	0.0547
**wR_2(F^2)**	0.3145	0.1368	0.1522
**GooF**	1.01	1.09	1.04
**2θ**	54	54	54
