# Supplementary figures and images for: Four- and five-component molecular solids: crystal engineering strategies based on structural inequivalence
Source: IUCrJ. 2016 Jan 5;3(Pt 2):96–101. doi: 10.1107/S2052252515023945 (PMC4775157; doi:10.1107/S2052252515023945)

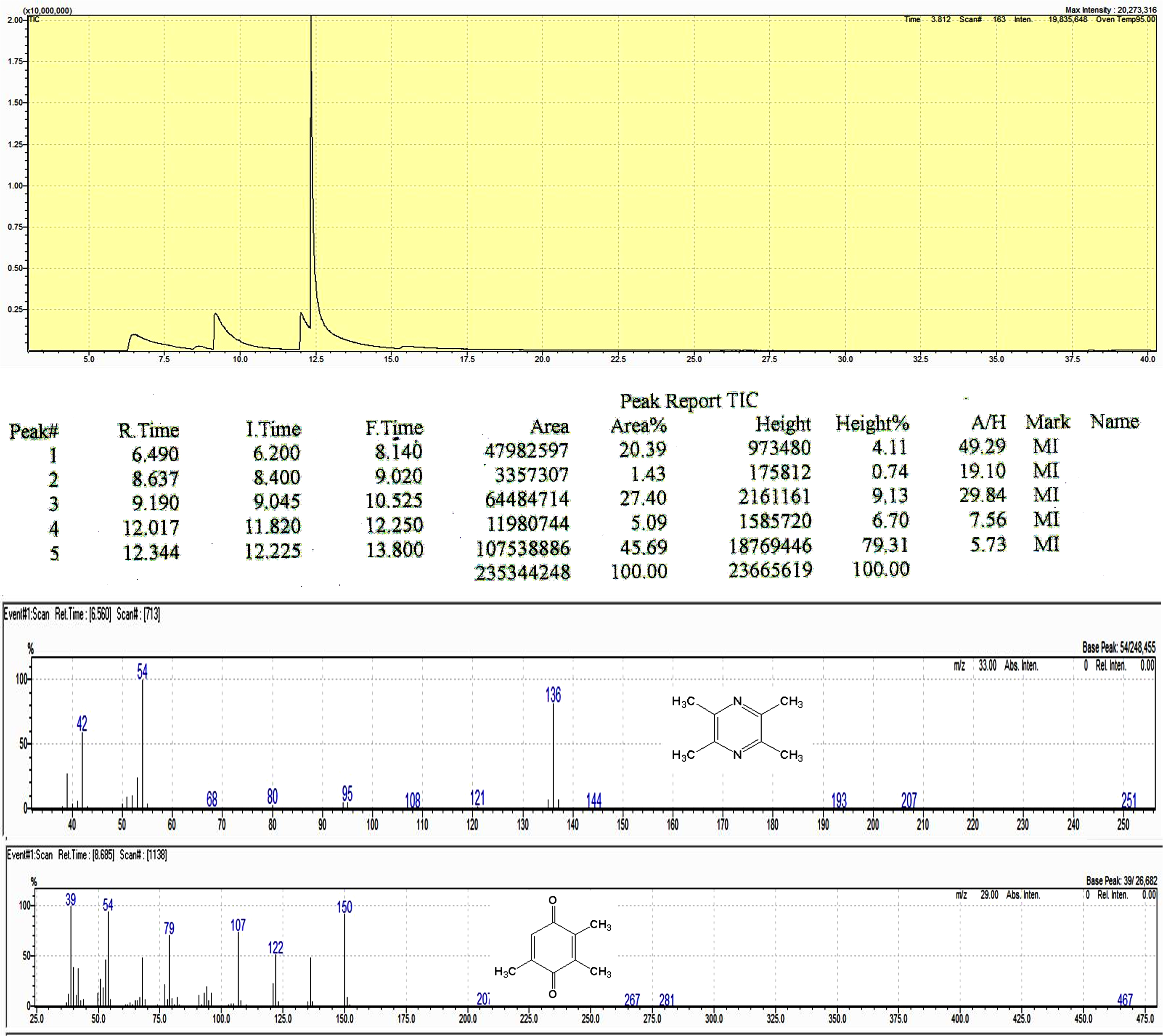

Supplement: Supplementary file 3 [file m-03-00096-sup3.zip › GC-MS/GC-MS a.tif]

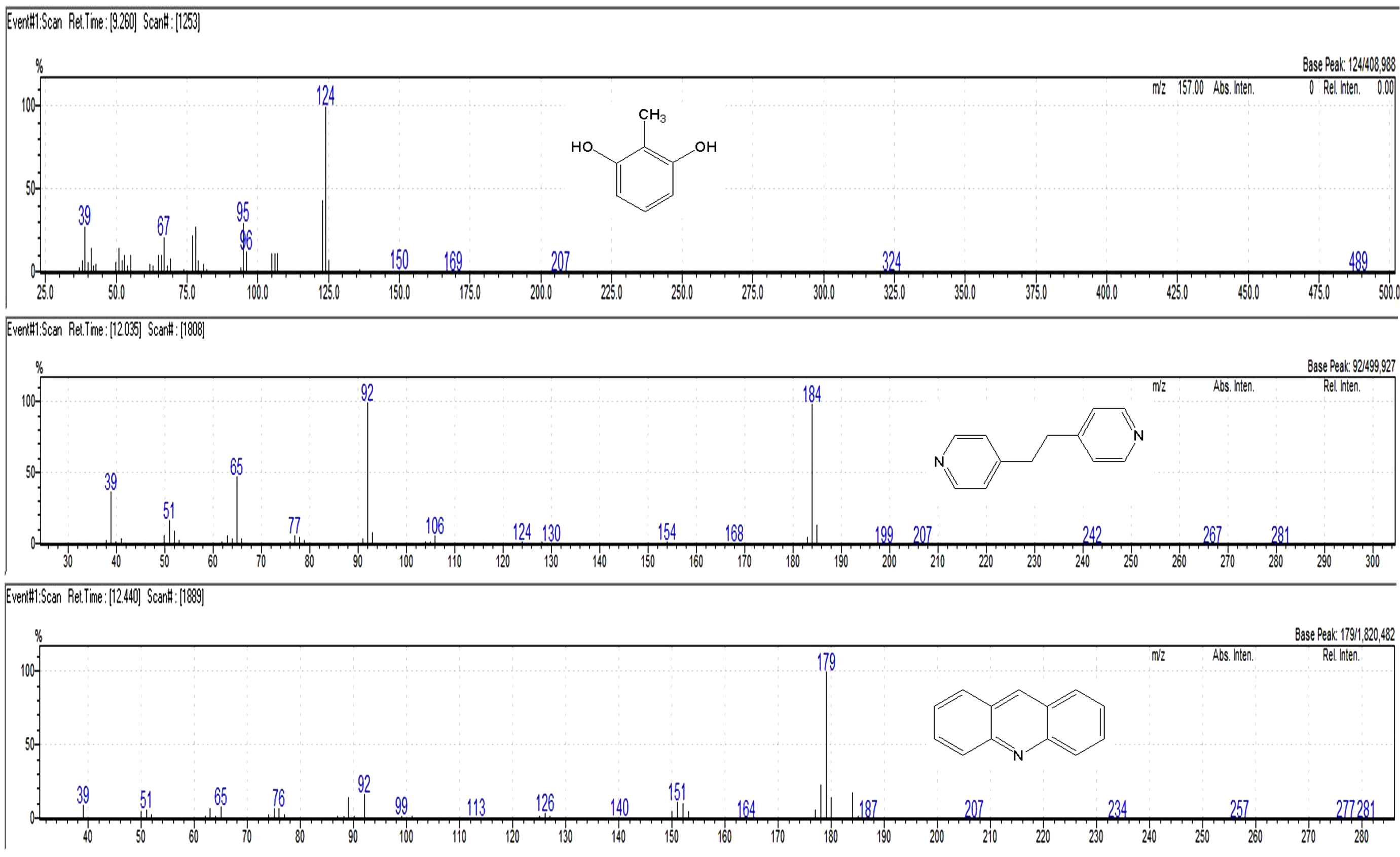

Supplement: Supplementary file 3 [file m-03-00096-sup3.zip › GC-MS/GC-MS b.tif]
