# Supplementary material for: Four- and five-component molecular solids: crystal engineering strategies based on structural inequivalence
Source: IUCrJ. 2016 Jan 5;3(Pt 2):96–101. doi: 10.1107/S2052252515023945 (PMC4775157; doi:10.1107/S2052252515023945)

checkCIF/PLATON report

You have not supplied any structure factors. As a result the full set of tests cannot be run.

THIS REPORT IS FOR GUIDANCE ONLY. IF USED AS PART OF A REVIEW PROCEDURE FOR PUBLICATION, IT SHOULD NOT REPLACE THE EXPERTISE OF AN EXPERIENCED CRYSTALLOGRAPHIC REFEREE.

No syntax errors found. CIF dictionary Interpreting this report

Datablock: shelx

```
Bond precision:  C-C = 0.0031 A          Wavelength=0.71073

Cell:           a=7.4709(3)       b=8.7326(3)      c=23.6381(9)
                alpha=92.857(1)   beta=91.317(1)   gamma=93.293(1)
Temperature:    293 K

                 Calculated                  Reported
Volume           1537.18(10)                 1537.18(10)
Space group      P -1                        P -1
Hall group       -P 1                        -P 1
                 C12 H12 N2, 2(C13 H9 N),
Moiety formula   C9 H9 O, 2(C8 H12 N2),      ?
                 2(C7 H8 O2)
Sum formula      C77 H79 N8 O5               C77 H79 N8 O5
Mr               1196.51                     1196.48
Dx,g cm-3        1.293                       1.292
Z                1                           1
Mu (mm-1)        0.082                       0.082
F000             637.0                       637.0
F000'            637.26
h,k,lmax         9,11,30                     9,11,30
Nref             7154                        7136
Tmin,Tmax        0.982,0.987                 0.982,0.987
Tmin'            0.957

Correction method= # Reported T Limits: Tmin=0.982 Tmax=0.987
AbsCorr = ANALYTICAL

Data completeness= 0.997          Theta(max)= 27.617

R(reflections)= 0.0593( 5177)     wR2(reflections)= 0.1643( 7136)

S = 1.031                 Npar= 432
```

The following ALERTS were generated. Each ALERT has the format
test-name_ALERT_alert-type_alert-level.
Click on the hyperlinks for more details of the test.

Alert level C

```
PLAT250_ALERT_2_C Large U3/U1 Ratio for Average U(i,j) Tensor ....        2.5 Note
PLAT250_ALERT_2_C Large U3/U1 Ratio for Average U(i,j) Tensor ....        2.2 Note
PLAT309_ALERT_2_C Single Bonded Oxygen (C-O > 1.3 Ang) ..........          O3 Check
PLAT413_ALERT_2_C Short Inter XH3 .. XHn     H28    ..  H38C    ..       2.12 Ang.
PLAT413_ALERT_2_C Short Inter XH3 .. XHn     H30    ..  H39A    ..       2.01 Ang.
PLAT790_ALERT_4_C Centre of Gravity not Within Unit Cell: Resd.  #          1 Note
              C12 H12 N2
```

Alert level G

```
PLAT007_ALERT_5_G Number of Unrefined Donor-H Atoms ..............          2 Report
PLAT066_ALERT_1_G Predicted and Reported Tmin&Tmax Range Identical          ? Check
PLAT154_ALERT_1_G The su's on the Cell Angles are Equal ..........    0.00100 Degree
PLAT199_ALERT_1_G Reported _cell_measurement_temperature ..... (K)        293 Check
PLAT200_ALERT_1_G Reported   _diffrn_ambient_temperature ..... (K)        293 Check
PLAT300_ALERT_4_G Atom Site Occupancy of *O3     is Constrained at      0.500 Check
PLAT300_ALERT_4_G Atom Site Occupancy of >C38    is Constrained at      0.720 Check
PLAT300_ALERT_4_G Atom Site Occupancy of >C39    is Constrained at      0.781 Check
PLAT300_ALERT_4_G Atom Site Occupancy of >H38A   is Constrained at      0.720 Check
PLAT300_ALERT_4_G Atom Site Occupancy of >H38B   is Constrained at      0.720 Check
PLAT300_ALERT_4_G Atom Site Occupancy of >H38C   is Constrained at      0.720 Check
PLAT300_ALERT_4_G Atom Site Occupancy of >H39A   is Constrained at      0.781 Check
PLAT300_ALERT_4_G Atom Site Occupancy of >H39B   is Constrained at      0.781 Check
PLAT300_ALERT_4_G Atom Site Occupancy of >H39C   is Constrained at      0.781 Check
PLAT301_ALERT_3_G Main Residue  Disorder ............ Percentage =          7 Note
PLAT432_ALERT_2_G Short Inter X...Y Contact  N2     ..  C38     ..       2.74 Ang.
PLAT432_ALERT_2_G Short Inter X...Y Contact  N2     ..  C39     ..       2.74 Ang.
PLAT790_ALERT_4_G Centre of Gravity not Within Unit Cell: Resd.  #          4 Note
              C8 H12 N2
PLAT790_ALERT_4_G Centre of Gravity not Within Unit Cell: Resd.  #          5 Note
              C7 H8 O2
```

---

0 **ALERT level A** = Most likely a serious problem - resolve or explain
0 **ALERT level B** = A potentially serious problem, consider carefully
6 **ALERT level C** = Check. Ensure it is not caused by an omission or oversight
19 **ALERT level G** = General information/check it is not something unexpected

4 ALERT type 1 CIF construction/syntax error, inconsistent or missing data
7 ALERT type 2 Indicator that the structure model may be wrong or deficient
1 ALERT type 3 Indicator that the structure quality may be low
12 ALERT type 4 Improvement, methodology, query or suggestion
1 ALERT type 5 Informative message, check

It is advisable to attempt to resolve as many as possible of the alerts in all categories. Often the minor alerts point to easily fixed oversights, errors and omissions in your CIF or refinement strategy, so attention to these fine details can be worthwhile. In order to resolve some of the more serious problems it may be necessary to carry out additional measurements or structure refinements. However, the purpose of your study may justify the reported deviations and the more serious of these should normally be commented upon in the discussion or experimental section of a paper or in the "special_details" fields of the CIF. checkCIF was carefully designed to identify outliers and unusual parameters, but every test has its limitations and alerts that are not important in a particular case may appear. Conversely, the absence of alerts does not guarantee there are no aspects of the results needing attention. It is up to the individual to critically assess their own results and, if necessary, seek expert advice.

Publication of your CIF in IUCr journals

A basic structural check has been run on your CIF. These basic checks will be run on all CIFs submitted for publication in IUCr journals (*Acta Crystallographica*, *Journal of Applied Crystallography*, *Journal of Synchrotron Radiation*); however, if you intend to submit to *Acta Crystallographica Section C* or *E*, you should make sure that full publication checks are run on the final version of your CIF prior to submission.

Publication of your CIF in other journals

Please refer to the *Notes for Authors* of the relevant journal for any special instructions relating to CIF submission.

PLATON version of 21/06/2015; check.def file version of 21/06/2015

Datablock shelx - ellipsoid plot

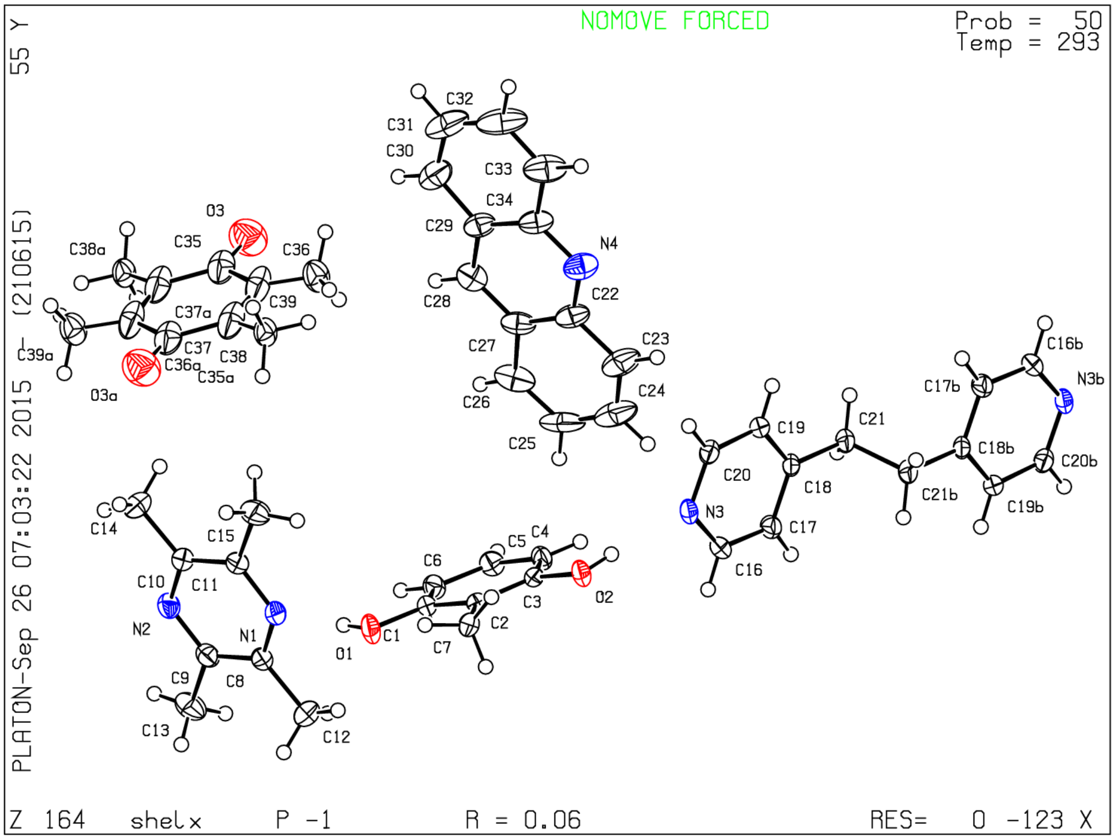

Supplement: Supplementary file 5 [file m-03-00096-sup6.zip › Checkcif Folder/Checkcif Reports/1. MRE TMP DPE-II ACR TMBQ.pdf]
