# Supplementary material for: Four- and five-component molecular solids: crystal engineering strategies based on structural inequivalence
Source: IUCrJ. 2016 Jan 5;3(Pt 2):96–101. doi: 10.1107/S2052252515023945 (PMC4775157; doi:10.1107/S2052252515023945)

checkCIF/PLATON report

You have not supplied any structure factors. As a result the full set of tests cannot be run.

THIS REPORT IS FOR GUIDANCE ONLY. IF USED AS PART OF A REVIEW PROCEDURE FOR PUBLICATION, IT SHOULD NOT REPLACE THE EXPERTISE OF AN EXPERIENCED CRYSTALLOGRAPHIC REFEREE.

No syntax errors found. CIF dictionary Interpreting this report

Datablock: shelx

Bond precision: C-C = 0.0024 A Wavelength=0.71073

Cell:	a=8.0059(6)	b=24.903(2)	c=12.0892(9)
	alpha=90	beta=91.065(3)	gamma=90
Temperature:	150 K		

	Calculated	Reported
Volume	2409.8(3)	2409.8(3)
Space group	P 21/c	P 21/c
Hall group	-P 2ybc	-P 2ybc
Moiety formula	C16 H10, C8 H12 N2, C7 H8 O2	?
Sum formula	C31 H30 N2 O2	C31 H30 N2 O2
Mr	462.57	462.57
Dx,g cm-3	1.275	1.275
Z	4	4
Mu (mm-1)	0.080	0.080
F000	984.0	984.0
F000’	984.39	
h,k,lmax	10,32,15	10,32,15
Nref	5535	5522
Tmin,Tmax	0.972,0.983	0.815,1.000
Tmin’	0.960	

Correction method= # Reported T Limits: Tmin=0.815 Tmax=1.000
AbsCorr = MULTI-SCAN

Data completeness= 0.998 Theta(max)= 27.496

R(reflections)= 0.0637(4725) wR2(reflections)= 0.1893(5522)

S = 1.096 Npar= 323

```
The following ALERTS were generated. Each ALERT has the format
       test-name_ALERT_alert-type_alert-level.
Click on the hyperlinks for more details of the test.
```

Alert level G

```
PLAT007_ALERT_5_G Number of Unrefined Donor-H Atoms ..............          2 Report
PLAT333_ALERT_2_G Check Large Av C6-Ring C-C Dist. C16    -C30          1.42 Ang.
PLAT333_ALERT_2_G Check Large Av C6-Ring C-C Dist. C20    -C30          1.42 Ang.
PLAT380_ALERT_4_G Incorrectly? Oriented X(sp2)-Methyl Moiety .....       C15 Check
```

```
   0 ALERT level A = Most likely a serious problem - resolve or explain
   0 ALERT level B = A potentially serious problem, consider carefully
   0 ALERT level C = Check. Ensure it is not caused by an omission or oversight
   4 ALERT level G = General information/check it is not something unexpected

   0 ALERT type 1 CIF construction/syntax error, inconsistent or missing data
   2 ALERT type 2 Indicator that the structure model may be wrong or deficient
   0 ALERT type 3 Indicator that the structure quality may be low
   1 ALERT type 4 Improvement, methodology, query or suggestion
   1 ALERT type 5 Informative message, check
```

It is advisable to attempt to resolve as many as possible of the alerts in all categories. Often the minor alerts point to easily fixed oversights, errors and omissions in your CIF or refinement strategy, so attention to these fine details can be worthwhile. In order to resolve some of the more serious problems it may be necessary to carry out additional measurements or structure refinements. However, the purpose of your study may justify the reported deviations and the more serious of these should normally be commented upon in the discussion or experimental section of a paper or in the "special_details" fields of the CIF. checkCIF was carefully designed to identify outliers and unusual parameters, but every test has its limitations and alerts that are not important in a particular case may appear. Conversely, the absence of alerts does not guarantee there are no aspects of the results needing attention. It is up to the individual to critically assess their own results and, if necessary, seek expert advice.

Publication of your CIF in IUCr journals

A basic structural check has been run on your CIF. These basic checks will be run on all CIFs submitted for publication in IUCr journals (*Acta Crystallographica*, *Journal of Applied Crystallography*, *Journal of Synchrotron Radiation*); however, if you intend to submit to *Acta Crystallographica Section C* or *E*, you should make sure that full publication checks are run on the final version of your CIF prior to submission.

Publication of your CIF in other journals

Please refer to the *Notes for Authors* of the relevant journal for any special instructions relating to CIF submission.

PLATON version of 21/06/2015; check.def file version of 21/06/2015

Datablock shelx - ellipsoid plot

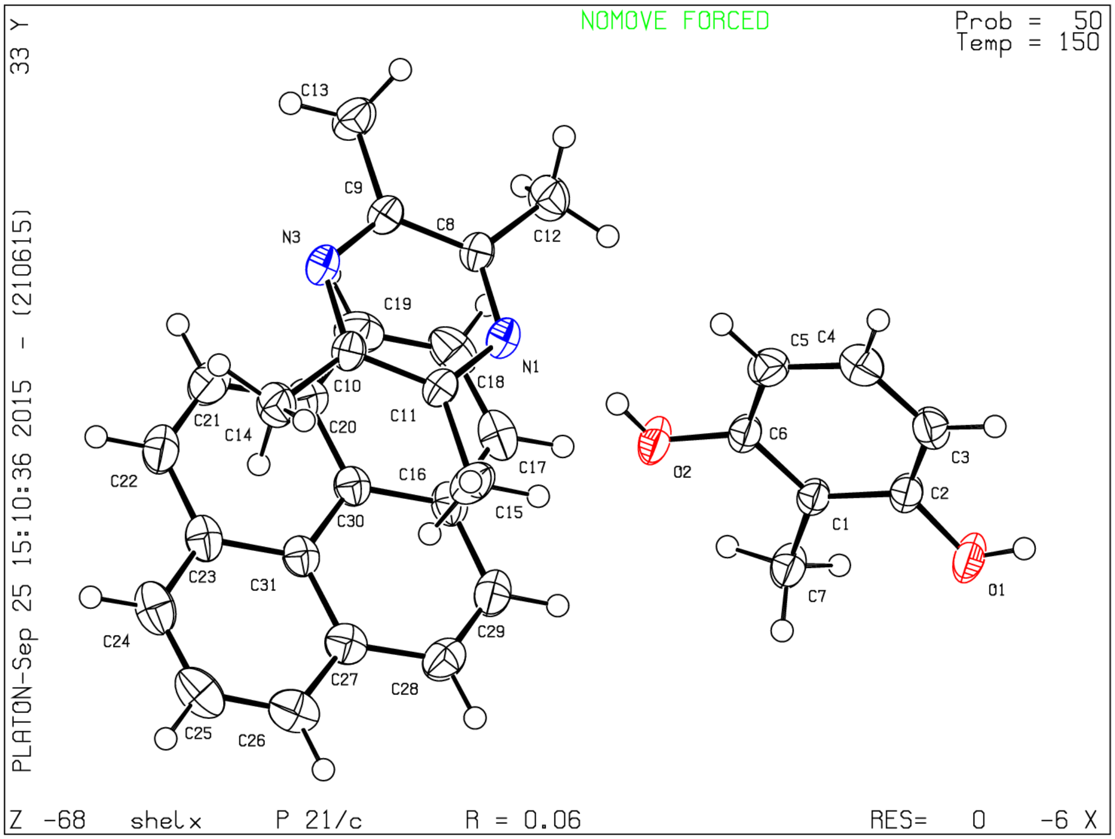

Supplement: Supplementary file 5 [file m-03-00096-sup6.zip › Checkcif Folder/Checkcif Reports/10. MRE TMP PYR.pdf]
