# Supplementary material for: Four- and five-component molecular solids: crystal engineering strategies based on structural inequivalence
Source: IUCrJ. 2016 Jan 5;3(Pt 2):96–101. doi: 10.1107/S2052252515023945 (PMC4775157; doi:10.1107/S2052252515023945)

checkCIF/PLATON report

You have not supplied any structure factors. As a result the full set of tests cannot be run.

THIS REPORT IS FOR GUIDANCE ONLY. IF USED AS PART OF A REVIEW PROCEDURE FOR PUBLICATION, IT SHOULD NOT REPLACE THE EXPERTISE OF AN EXPERIENCED CRYSTALLOGRAPHIC REFEREE.

No syntax errors found. CIF dictionary Interpreting this report

Datablock: shelx

```
Bond precision:  C-C = 0.0032 A          Wavelength=0.71073

Cell:          a=7.4914(3)       b=8.8265(4)      c=23.583(1)
               alpha=92.722(1)   beta=92.976(1)   gamma=91.626(1)
Temperature:   150 K
```

	Calculated	Reported
Volume	1554.77(11)	1554.77(11)
Space group	P -1	P -1
Hall group	-P 1	-P 1
Moiety formula	C12 H12 N2, 2(C14 H10), C7 H5 O2, 2(C8 H12 N2), 2(C7 H8 O2)	?
Sum formula	C77 H77 N6 O6	C77 H77 N6 O6
Mr	1182.45	1182.44
Dx,g cm-3	1.263	1.263
Z	1	1
Mu (mm-1)	0.080	0.080
F000	629.0	629.0
F000’	629.25	
h,k,lmax	9,10,29	9,10,29
Nref	6106	6096
Tmin,Tmax	0.963,0.983	0.963,0.983
Tmin’	0.932	

```
Correction method= # Reported T Limits: Tmin=0.963 Tmax=0.983
AbsCorr = ANALYTICAL

Data completeness= 0.998          Theta(max)= 25.996

R(reflections)= 0.0545( 4165)     wR2(reflections)= 0.1641( 6096)

S = 1.059                  Npar= 433
```

The following ALERTS were generated. Each ALERT has the format
test-name_ALERT_alert-type_alert-level.
Click on the hyperlinks for more details of the test.

Alert level C

```
PLAT213_ALERT_2_C Atom C26            has ADP max/min Ratio .....        3.3 prolat
PLAT250_ALERT_2_C Large U3/U1 Ratio for Average U(i,j) Tensor ....       3.2 Note
PLAT250_ALERT_2_C Large U3/U1 Ratio for Average U(i,j) Tensor ....       3.8 Note
PLAT790_ALERT_4_C Centre of Gravity not Within Unit Cell: Resd.  #         1 Note
              C12 H12 N2
```

Alert level G

```
PLAT003_ALERT_2_G Number of Uiso or Uij Restrained non-H Atoms ...         8 Report
PLAT007_ALERT_5_G Number of Unrefined Donor-H Atoms ..............         4 Report
PLAT063_ALERT_4_G Crystal Size Likely too Large for Beam Size ....      0.88 mm
PLAT066_ALERT_1_G Predicted and Reported Tmin&Tmax Range Identical         ? Check
PLAT154_ALERT_1_G The su's on the Cell Angles are Equal ..........   0.00100 Degree
PLAT177_ALERT_4_G The CIF-Embedded .res File Contains DELU Records         1 Report
PLAT300_ALERT_4_G Atom Site Occupancy of *O3     is Constrained at     0.500 Check
PLAT300_ALERT_4_G Atom Site Occupancy of *O4     is Constrained at     0.500 Check
PLAT300_ALERT_4_G Atom Site Occupancy of *C40    is Constrained at     0.500 Check
PLAT300_ALERT_4_G Atom Site Occupancy of *H3A    is Constrained at     0.500 Check
PLAT300_ALERT_4_G Atom Site Occupancy of *H4     is Constrained at     0.500 Check
PLAT300_ALERT_4_G Atom Site Occupancy of *H40A   is Constrained at     0.500 Check
PLAT300_ALERT_4_G Atom Site Occupancy of *H40B   is Constrained at     0.500 Check
PLAT300_ALERT_4_G Atom Site Occupancy of *H40C   is Constrained at     0.500 Check
PLAT301_ALERT_3_G Main Residue  Disorder ............ Percentage =         5 Note
PLAT380_ALERT_4_G Incorrectly? Oriented X(sp2)-Methyl Moiety .....       C14 Check
PLAT860_ALERT_3_G Number of Least-Squares Restraints .............        10 Note
```

```
   0 ALERT level A = Most likely a serious problem - resolve or explain
   0 ALERT level B = A potentially serious problem, consider carefully
   4 ALERT level C = Check. Ensure it is not caused by an omission or oversight
  17 ALERT level G = General information/check it is not something unexpected

   2 ALERT type 1 CIF construction/syntax error, inconsistent or missing data
   4 ALERT type 2 Indicator that the structure model may be wrong or deficient
   2 ALERT type 3 Indicator that the structure quality may be low
  12 ALERT type 4 Improvement, methodology, query or suggestion
   1 ALERT type 5 Informative message, check
```

It is advisable to attempt to resolve as many as possible of the alerts in all categories. Often the minor alerts point to easily fixed oversights, errors and omissions in your CIF or refinement strategy, so attention to these fine details can be worthwhile. In order to resolve some of the more serious problems it may be necessary to carry out additional measurements or structure refinements. However, the purpose of your study may justify the reported deviations and the more serious of these should normally be commented upon in the discussion or experimental section of a paper or in the "special_details" fields of the CIF. checkCIF was carefully designed to identify outliers and unusual parameters, but every test has its limitations and alerts that are not important in a particular case may appear. Conversely, the absence of alerts does not guarantee there are no aspects of the results needing attention. It is up to the individual to critically assess their own results and, if necessary, seek expert advice.

Publication of your CIF in IUCr journals

A basic structural check has been run on your CIF. These basic checks will be run on all CIFs submitted for publication in IUCr journals (*Acta Crystallographica*, *Journal of Applied Crystallography*, *Journal of Synchrotron Radiation*); however, if you intend to submit to *Acta Crystallographica Section C* or *E*, you should make sure that full publication checks are run on the final version of your CIF prior to submission.

Publication of your CIF in other journals

Please refer to the *Notes for Authors* of the relevant journal for any special instructions relating to CIF submission.

PLATON version of 21/06/2015; check.def file version of 21/06/2015

Datablock shelx - ellipsoid plot

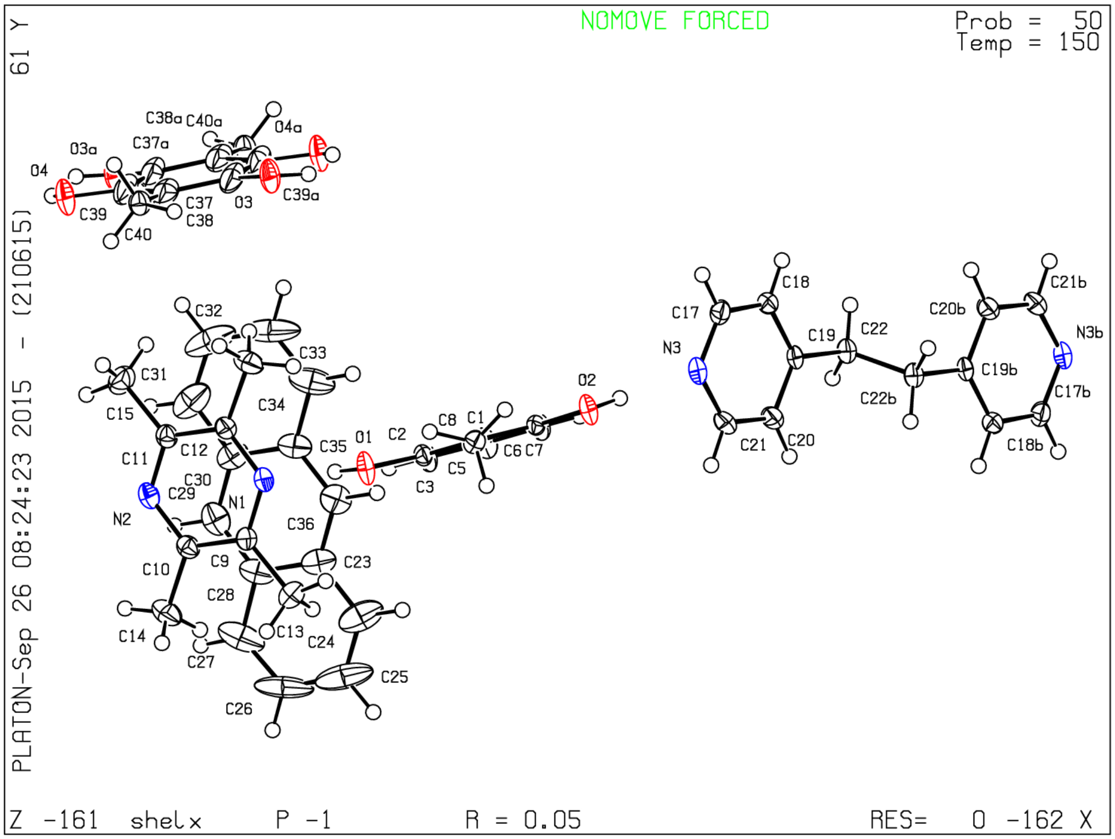

Supplement: Supplementary file 5 [file m-03-00096-sup6.zip › Checkcif Folder/Checkcif Reports/17. MRE TMP DPE-II ANT.pdf]
