# Supplementary material for: Four- and five-component molecular solids: crystal engineering strategies based on structural inequivalence
Source: IUCrJ. 2016 Jan 5;3(Pt 2):96–101. doi: 10.1107/S2052252515023945 (PMC4775157; doi:10.1107/S2052252515023945)

checkCIF/PLATON report

You have not supplied any structure factors. As a result the full set of tests cannot be run.

THIS REPORT IS FOR GUIDANCE ONLY. IF USED AS PART OF A REVIEW PROCEDURE FOR PUBLICATION, IT SHOULD NOT REPLACE THE EXPERTISE OF AN EXPERIENCED CRYSTALLOGRAPHIC REFEREE.

No syntax errors found. CIF dictionary Interpreting this report

Datablock: shelx

```
Bond precision:  C-C = 0.0054 A         Wavelength=0.71073

Cell:          a=7.478(12)      b=8.739(14)     c=23.37(3)
               alpha=92.36(3)   beta=90.29(3)   gamma=93.131(19)
Temperature:   150 K

                Calculated                 Reported
Volume          1524(4)                    1524(4)
Space group     P -1                       P -1
Hall group      -P 1                       -P 1
                2(C13 H9 N), C12 H10 N2,
Moiety formula  C7 H5 O2, 2(C8 H12 N2),    ?
                2(C7 H8 O2)
Sum formula     C75 H73 N8 O6              C75 H73 N8 O6
Mr              1182.41                    1182.41
Dx,g cm-3       1.288                      1.288
Z               1                          1
Mu (mm-1)       0.083                      0.083
F000            627.0                      627.0
F000'           627.25
h,k,lmax        9,11,30                    9,11,30
Nref            7304                       7022
Tmin,Tmax       0.975,0.990                0.532,1.000
Tmin'           0.975

Correction method= # Reported T Limits: Tmin=0.532 Tmax=1.000
AbsCorr = MULTI-SCAN

Data completeness= 0.961          Theta(max)= 27.892

R(reflections)= 0.0922( 4024)     wR2(reflections)= 0.3001( 7022)

S = 1.011                   Npar= 418
```

The following ALERTS were generated. Each ALERT has the format
test-name_ALERT_alert-type_alert-level.
Click on the hyperlinks for more details of the test.

Alert level A

```
PLAT312_ALERT_2_A Strange C-O-H Geometry (C-O  <   1.25 Ang) .....         O4 Check
```

Alert level C

```
RFACR01_ALERT_3_C  The value of the weighted R factor is > 0.25
            Weighted R factor given   0.300
PLAT029_ALERT_3_C _diffrn_measured_fraction_theta_full Low .......      0.974 Note
PLAT084_ALERT_3_C High wR2 Value (i.e. > 0.25) ...................       0.30 Report
PLAT148_ALERT_3_C su on the       a  - Axis is (Too) Large ........      0.012 Ang.
PLAT148_ALERT_3_C su on the       b  - Axis is (Too) Large ........      0.014 Ang.
PLAT148_ALERT_3_C su on the       c  - Axis is (Too) Large ........      0.030 Ang.
PLAT250_ALERT_2_C Large U3/U1 Ratio for Average U(i,j) Tensor ....        2.7 Note
PLAT340_ALERT_3_C Low Bond Precision on  C-C Bonds ...............     0.0054 Ang.
```

Alert level G

```
PLAT007_ALERT_5_G Number of Unrefined Donor-H Atoms ..............          4 Report
PLAT072_ALERT_2_G SHELXL First  Parameter in WGHT Unusually Large.       0.16 Report
PLAT300_ALERT_4_G Atom Site Occupancy of *O3     is Constrained at      0.500 Check
PLAT300_ALERT_4_G Atom Site Occupancy of *O4     is Constrained at      0.500 Check
PLAT300_ALERT_4_G Atom Site Occupancy of *C19    is Constrained at      0.500 Check
PLAT300_ALERT_4_G Atom Site Occupancy of *H3A    is Constrained at      0.500 Check
PLAT300_ALERT_4_G Atom Site Occupancy of *H4A    is Constrained at      0.500 Check
PLAT300_ALERT_4_G Atom Site Occupancy of *H19A   is Constrained at      0.500 Check
PLAT300_ALERT_4_G Atom Site Occupancy of *H19B   is Constrained at      0.500 Check
PLAT300_ALERT_4_G Atom Site Occupancy of *H19C   is Constrained at      0.500 Check
PLAT301_ALERT_3_G Main Residue  Disorder ............ Percentage =          5 Note
PLAT790_ALERT_4_G Centre of Gravity not Within Unit Cell: Resd.  #          2 Note
              C12 H10 N2
```

1 **ALERT level A** = Most likely a serious problem - resolve or explain
0 **ALERT level B** = A potentially serious problem, consider carefully
8 **ALERT level C** = Check. Ensure it is not caused by an omission or oversight
12 **ALERT level G** = General information/check it is not something unexpected

0 ALERT type 1 CIF construction/syntax error, inconsistent or missing data
3 ALERT type 2 Indicator that the structure model may be wrong or deficient
8 ALERT type 3 Indicator that the structure quality may be low
9 ALERT type 4 Improvement, methodology, query or suggestion
1 ALERT type 5 Informative message, check

It is advisable to attempt to resolve as many as possible of the alerts in all categories. Often the minor alerts point to easily fixed oversights, errors and omissions in your CIF or refinement strategy, so attention to these fine details can be worthwhile. In order to resolve some of the more serious problems it may be necessary to carry out additional measurements or structure refinements. However, the purpose of your study may justify the reported deviations and the more serious of these should normally be commented upon in the discussion or experimental section of a paper or in the "special_details" fields of the CIF. checkCIF was carefully designed to identify outliers and unusual parameters, but every test has its limitations and alerts that are not important in a particular case may appear. Conversely, the absence of alerts does not guarantee there are no aspects of the results needing attention. It is up to the individual to critically assess their own results and, if necessary, seek expert advice.

Publication of your CIF in IUCr journals

A basic structural check has been run on your CIF. These basic checks will be run on all CIFs submitted for publication in IUCr journals (*Acta Crystallographica*, *Journal of Applied Crystallography*, *Journal of Synchrotron Radiation*); however, if you intend to submit to *Acta Crystallographica Section C* or *E*, you should make sure that full publication checks are run on the final version of your CIF prior to submission.

Publication of your CIF in other journals

Please refer to the *Notes for Authors* of the relevant journal for any special instructions relating to CIF submission.

PLATON version of 21/06/2015; check.def file version of 21/06/2015

Datablock shelx - ellipsoid plot

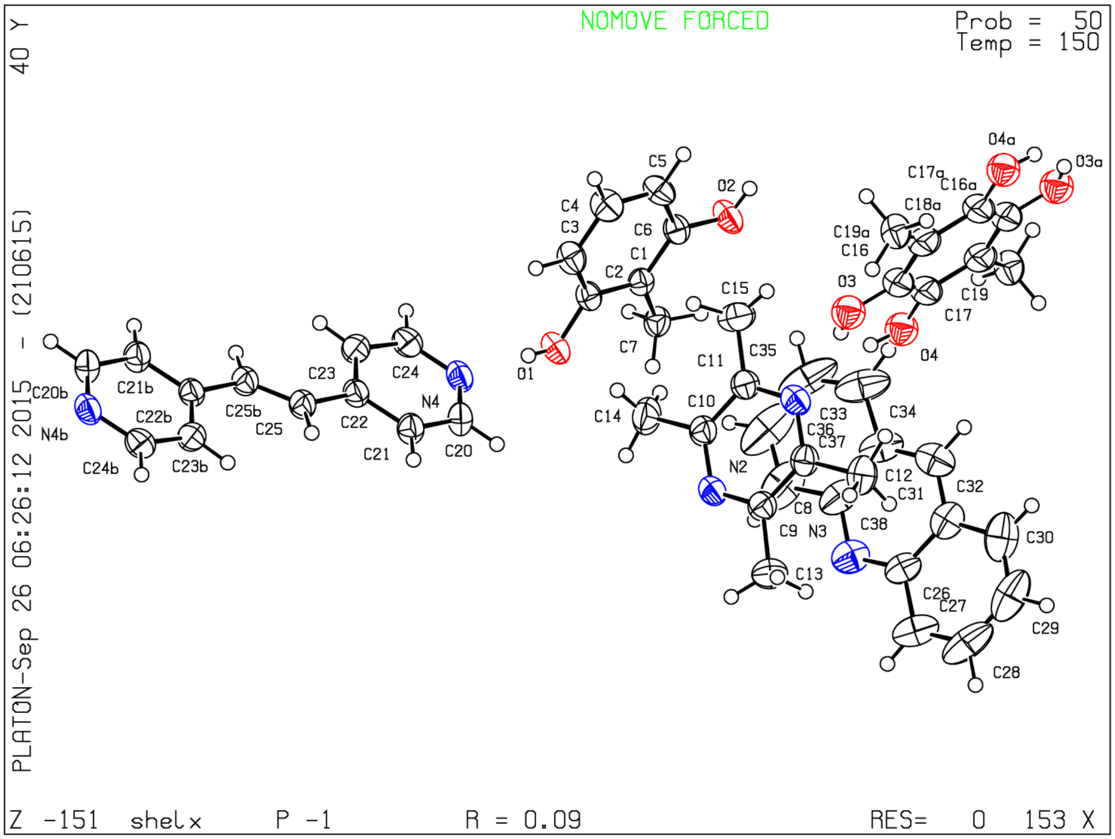

Supplement: Supplementary file 5 [file m-03-00096-sup6.zip › Checkcif Folder/Checkcif Reports/19. MRE TMP DPE-I ACR Form II.pdf]
