# Supplementary material for: Four- and five-component molecular solids: crystal engineering strategies based on structural inequivalence
Source: IUCrJ. 2016 Jan 5;3(Pt 2):96–101. doi: 10.1107/S2052252515023945 (PMC4775157; doi:10.1107/S2052252515023945)

checkCIF/PLATON report

You have not supplied any structure factors. As a result the full set of tests cannot be run.

THIS REPORT IS FOR GUIDANCE ONLY. IF USED AS PART OF A REVIEW PROCEDURE FOR PUBLICATION, IT SHOULD NOT REPLACE THE EXPERTISE OF AN EXPERIENCED CRYSTALLOGRAPHIC REFEREE.

No syntax errors found. CIF dictionary Interpreting this report

Datablock: shelx

```
Bond precision:  C-C = 0.0026 A          Wavelength=0.71073

Cell:          a=7.4611(3)       b=8.7285(4)      c=23.6219(10)
               alpha=92.850(1)   beta=91.347(1)   gamma=93.317(1)
Temperature:   150 K

                Calculated                Reported
Volume          1533.34(11)               1533.34(11)
Space group     P -1                      P -1
Hall group      -P 1                      -P 1
                C12 H12 N2, 2(C13 H9 N),
Moiety formula  C7 H5 O2, 2(C8 H12 N2),   ?
                2(C7 H8 O2)
Sum formula     C75 H75 N8 O6             C75 H75 N8 O6
Mr              1184.43                   1184.43
Dx,g cm-3       1.283                     1.283
Z               1                         1
Mu (mm-1)       0.082                     0.082
F000            629.0                     629.0
F000'           629.25
h,k,lmax        9,11,30                   9,11,30
Nref            7082                      7056
Tmin,Tmax       0.969,0.981               0.969,0.981
Tmin'           0.951

Correction method= # Reported T Limits: Tmin=0.969 Tmax=0.981
AbsCorr = ANALYTICAL

Data completeness= 0.996           Theta(max)= 27.545

R(reflections)= 0.0552( 6163)      wR2(reflections)= 0.1356( 7056)

S = 1.091                  Npar= 434
```

The following ALERTS were generated. Each ALERT has the format
test-name_ALERT_alert-type_alert-level.
Click on the hyperlinks for more details of the test.

Alert level C

```
PLAT250_ALERT_2_C Large U3/U1 Ratio for Average U(i,j) Tensor ....        2.5 Note
PLAT250_ALERT_2_C Large U3/U1 Ratio for Average U(i,j) Tensor ....        2.7 Note
```

Alert level G

```
PLAT007_ALERT_5_G Number of Unrefined Donor-H Atoms ..............          4 Report
PLAT063_ALERT_4_G Crystal Size Likely too Large for Beam Size ....       0.61 mm
PLAT066_ALERT_1_G Predicted and Reported Tmin&Tmax Range Identical          ? Check
PLAT154_ALERT_1_G The su's on the Cell Angles are Equal ..........    0.00100 Degree
PLAT300_ALERT_4_G Atom Site Occupancy of *O3     is Constrained at      0.500 Check
PLAT300_ALERT_4_G Atom Site Occupancy of *O4     is Constrained at      0.500 Check
PLAT300_ALERT_4_G Atom Site Occupancy of *C36    is Constrained at      0.500 Check
PLAT300_ALERT_4_G Atom Site Occupancy of *H3A    is Constrained at      0.500 Check
PLAT300_ALERT_4_G Atom Site Occupancy of *H4A    is Constrained at      0.500 Check
PLAT300_ALERT_4_G Atom Site Occupancy of *H36A   is Constrained at      0.500 Check
PLAT300_ALERT_4_G Atom Site Occupancy of *H36B   is Constrained at      0.500 Check
PLAT300_ALERT_4_G Atom Site Occupancy of *H36C   is Constrained at      0.500 Check
PLAT301_ALERT_3_G Main Residue  Disorder ............ Percentage =          5 Note
PLAT432_ALERT_2_G Short Inter X...Y Contact  C36    ..  C36     ..       3.19 Ang.
PLAT790_ALERT_4_G Centre of Gravity not Within Unit Cell: Resd.  #          4 Note
              C8 H12 N2
PLAT790_ALERT_4_G Centre of Gravity not Within Unit Cell: Resd.  #          5 Note
              C7 H8 O2
```

---

0 **ALERT level A** = Most likely a serious problem - resolve or explain
0 **ALERT level B** = A potentially serious problem, consider carefully
2 **ALERT level C** = Check. Ensure it is not caused by an omission or oversight
16 **ALERT level G** = General information/check it is not something unexpected

2 ALERT type 1 CIF construction/syntax error, inconsistent or missing data
3 ALERT type 2 Indicator that the structure model may be wrong or deficient
1 ALERT type 3 Indicator that the structure quality may be low
11 ALERT type 4 Improvement, methodology, query or suggestion
1 ALERT type 5 Informative message, check

It is advisable to attempt to resolve as many as possible of the alerts in all categories. Often the minor alerts point to easily fixed oversights, errors and omissions in your CIF or refinement strategy, so attention to these fine details can be worthwhile. In order to resolve some of the more serious problems it may be necessary to carry out additional measurements or structure refinements. However, the purpose of your study may justify the reported deviations and the more serious of these should normally be commented upon in the discussion or experimental section of a paper or in the "special_details" fields of the CIF. checkCIF was carefully designed to identify outliers and unusual parameters, but every test has its limitations and alerts that are not important in a particular case may appear. Conversely, the absence of alerts does not guarantee there are no aspects of the results needing attention. It is up to the individual to critically assess their own results and, if necessary, seek expert advice.

Publication of your CIF in IUCr journals

A basic structural check has been run on your CIF. These basic checks will be run on all CIFs submitted for publication in IUCr journals (*Acta Crystallographica*, *Journal of Applied Crystallography*, *Journal of Synchrotron Radiation*); however, if you intend to submit to *Acta Crystallographica Section C* or *E*, you should make sure that full publication checks are run on the final version of your CIF prior to submission.

Publication of your CIF in other journals

Please refer to the *Notes for Authors* of the relevant journal for any special instructions relating to CIF submission.

PLATON version of 21/06/2015; check.def file version of 21/06/2015

Datablock shelx - ellipsoid plot

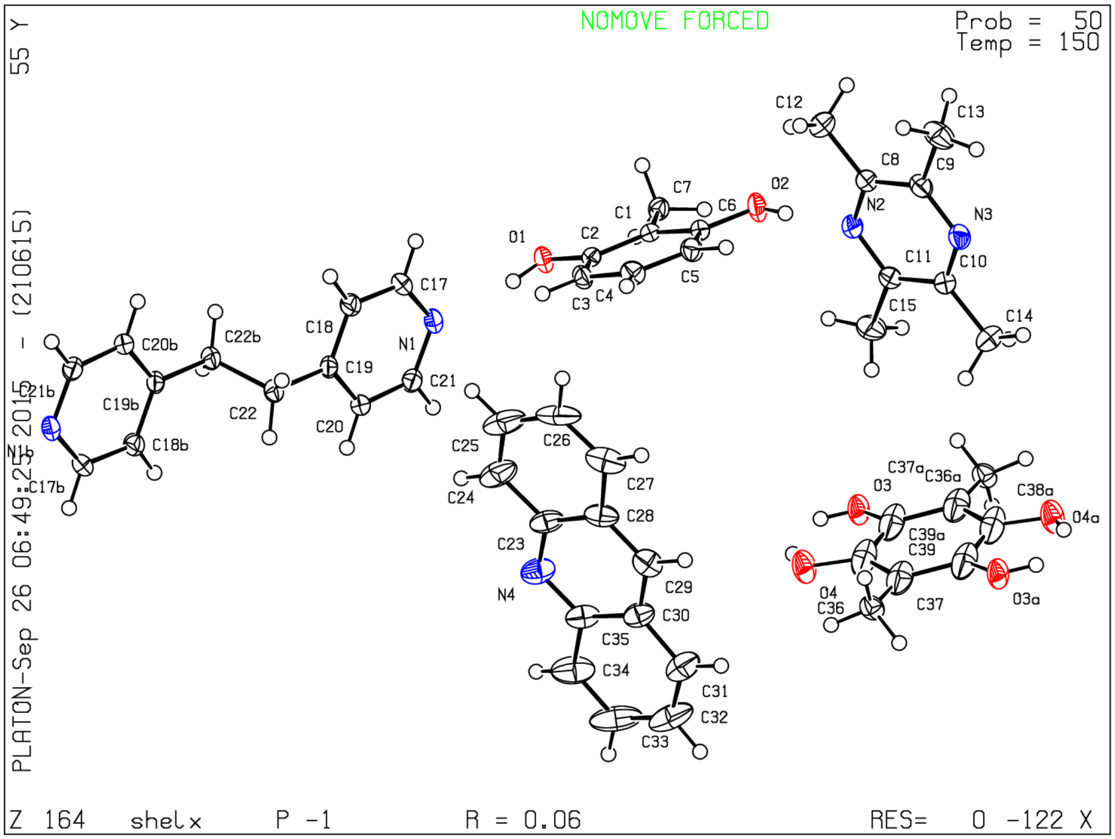

Supplement: Supplementary file 5 [file m-03-00096-sup6.zip › Checkcif Folder/Checkcif Reports/20. MRE TMP DPE-II ACR.pdf]
